# Robust classification of protein variation using structural modelling and large-scale data integration

**DOI:** 10.1093/nar/gkw120

**Published:** 2016-02-28

**Authors:** Evan H. Baugh, Riley Simmons-Edler, Christian L. Müller, Rebecca F. Alford, Natalia Volfovsky, Alex E. Lash, Richard Bonneau

**Affiliations:** 1 Department of Biology, New York University, New York, NY 10003, USA; 2 New York University Center for Genomics and Systems Biology, New York, NY 10003, USA; 3 Computer Science Department, New York University, New York, NY 10003, USA; 4 Simons Center for Data Analysis, Simons Foundation, New York, NY 10010, USA; 5 Carnegie Mellon University Department of Chemistry, 5000 Forbes Ave, Pittsburgh, PA 15289, USA; 6 Commack High School, Commack, NY 11725, USA; 7 Simons Foundation, New York, NY 10010, USA

## Abstract

Existing methods for interpreting protein variation focus on annotating mutation pathogenicity rather than detailed interpretation of variant deleteriousness and frequently use only sequence-based or structure-based information. We present VIPUR, a computational framework that seamlessly integrates sequence analysis and structural modelling (using the Rosetta protein modelling suite) to identify and interpret deleterious protein variants. To train VIPUR, we collected 9477 protein variants with known effects on protein function from multiple organisms and curated structural models for each variant from crystal structures and homology models. VIPUR can be applied to mutations in any organism's proteome with improved generalized accuracy (AUROC .83) and interpretability (AUPR .87) compared to other methods. We demonstrate that VIPUR's predictions of deleteriousness match the biological phenotypes in ClinVar and provide a clear ranking of prediction confidence. We use VIPUR to interpret known mutations associated with inflammation and diabetes, demonstrating the structural diversity of disrupted functional sites and improved interpretation of mutations associated with human diseases. Lastly, we demonstrate VIPUR's ability to highlight candidate variants associated with human diseases by applying VIPUR to *de novo* variants associated with autism spectrum disorders.

## INTRODUCTION

High-throughput sequencing technologies and new computational techniques for analyzing population genetics data are rapidly improving our understanding of disease susceptibility in humans (1–3) and adaptation in a wide variety of organisms, including crop species and pathogens (4–6). These studies often discover nonsynonymous variation with large effects as even a single amino acid change can disrupt the folding, catalytic activity and physical interactions of proteins (7,8). Current estimates predict that every human genome contains 10,000–11,000 nonsynonymous variations (9,10) and, while we cannot currently characterize all this diversity experimentally, many variants that alter protein function can be identified computationally from destabilization of structural models or amino acid conservation (4,11–12). Methods for annotating variant effects in genome-wide association studies and exome sequencing studies, such as PolyPhen2 (13), CADD (14), PROVEAN (15) and SIFT (16), use conservation and other sequence-based features to identify damaging variants but cannot predict the effects these variants have on protein function. Recent studies of *de novo* variants (17–19) have demonstrated the power of these methods but also the need for additional information (4), such as physical models from the Protein Data Bank (PDB) (20), to identify causal variants in disease association studies.

Most methods for annotating coding variants attempt to predict variant deleteriousness in the context of the whole organism (where deleteriousness is defined as the tendency for a variant to reduce organismal fitness, to express an altered phenotype or to exhibit an association with a disease condition) (14). Deleteriousness, when defined in terms of fitness or phenotypic effects, is difficult to measure directly but underlies patterns of conservation, molecular functionality and disease pathogenicity. Variant annotations in several databases are often limited to discrete labels such as deleterious or neutral. Definitions based on deleteriousness are often confused with definitions of pathogenicity used to curate training and benchmarking on datasets. The annotations predicted by current coding variant annotation methods for these reasons have diverse implications. For example, SIFT segregates ‘tolerant’ from ‘intolerant’ variants (16), while PolyPhen2 identifies ‘possibly damaging’ and ‘probably damaging’ effects (13). CADD predicts deleteriousness by distinguishing fixed from simulated variation and relies on the predictions of other methods including both SIFT and PolyPhen2 (14). Each of these methods predicts a label that is designed to correlate with variant deleteriousness and is used to prioritize causal pathogenic variants from large genomic datasets (4). Variant annotation methods are used to identify variants with large effects on disease phenotypes and despite being trained for slightly different purposes, they can be compared by their ability to prioritize candidate variants.

Deleteriousness can be approximated with measures of conservation and molecular functionality but available data on both protein sequence variation and structural energetics are rarely combined (21–23). Selection against deleterious variants can be detected by analysis of conservation and other alignment-based methods, although these metrics may not apply to *de novo* mutations. Alternatively, several studies have aimed to model the biophysical characteristics of mutations, such as energetic stability, enzymatic function and the *pK*_*a*_ of key residues. Protein structure models of mutations can be used to indicate disruption of active sites and destabilization of the folded protein (7,21,24–25) using tools like Rosetta (25,26) and FoldX (24). Here we aim to provide a measure of deleteriousness centred on individual proteins, with our deleterious label indicating disrupted protein function (disrupted stability, active site, interface or folding). Our method aims to use conservation and structural analyses to better predict protein-centred deleteriousness.

We present **VIPUR** (*vipƏ(r)*, **V**ariant **I**nterpretation and **P**rediction **U**sing **R**osetta), a computational framework capable of identifying, ranking and interpreting deleterious protein variants in different species. To make VIPUR applicable across multiple species, we curated **VTS** (the **V**IPUR **T**raining **S**et), a novel collection of 9,477 annotated variants from >360 species containing both natural variations and experimental mutations. Variant annotations were carefully curated, restricting **VTS** to deleterious variants which directly disrupt protein molecular function or are functionally neutral, rather than ‘pathogenicity’ or ‘intolerance’. We obtained structural models for these proteins from solved crystal structures and comparative modelling initiatives, such as ModBase (27), taking advantage of reliable homology models freely available for most human proteins. Structural analysis is performed using Rosetta to rigorously sample variant protein conformations, properly accommodating the variant amino acid by moving the protein backbone (23,25,28). We combine sequence-based and structure-based features in a sparse logistic regression framework, leading to a classifier that accurately ranks deleterious variants, with ≥90% precision on the highest scoring 3,800 variants (40% of variants classified) and 0.872 Area Under the Precision-Recall curve (AUPR). In addition to classification and ranking, VIPUR uses structural analysis to provide a detailed prediction of each variant's physical effect, automatically reporting disruption of hydrogen bonding, side-chain packing and backbone stability.

VIPUR deleterious predictions do not guarantee the presence of a disease phenotype. Nonetheless, distributions of VIPUR scores match expectations for known pathogenic and benign variant phenotypes in ClinVar (29) with deleterious predictions enriched for pathogenic variants and neutral predictions enriched for benign variants. We apply VIPUR to a small set of variants (388) in proteins associated with inflammation and diabetes mellitus to identify deleterious variants improperly annotated by sequence-based methods and demonstrate the clarity of VIPUR predictions. We demonstrate the ability of VIPUR to identify and rank potentially causal variants in the *de novo* missense mutations of the Simons Simplex Collection (SSC) (30–32) and compare to other variant annotation methods (2,226 missense variants). While the stated goals of these methods differ, they are all used in practice to prioritize variants and genes for future investigation. VIPUR deleterious predictions demonstrate a clear enrichment for mutations found in children with autism that is unmatched by current variant annotation methods and highlights a small set of extremely confident candidate variants for future investigation.

## MATERIALS AND METHODS

### Generating a deleterious protein variant benchmark

Existing datasets for the training and benchmarking of protein variant annotation methods are frequently restricted in scope, focusing on disease-associated variants (13,15,33–34). Methods that model protein structures are similarly restricted, validating on *in vitro* experimental characterization of variants produced by mutagenesis (21,24). We want VIPUR to predict variant deleteriousness and generalize to both natural variants and mutagenesis variants. We collected and curated missense variants from multiple experimental sources and prepared structural models from different databases to ensure VIPUR is benchmarked on diverse protein structures (see Supplementary Figure S1). Protein variants from HumDiv (13) and UniProt (35) with clear ‘deleterious’ or ‘neutral’ effects were mapped onto crystallographic and comparative models of the protein macromolecules from the PDB (20), ModBase (27) and SwissModel (36). Our deleterious and neutral labels are restricted to variants with direct evidence of protein disruption, avoiding the assumptions that all disease-associated variants are necessarily deleterious (13) or that all unannotated variants are necessarily neutral (15). This training set, **VTS**, includes 9,477 variants (5,740 deleterious, 3,737 neutral, 1.54 label ratio) and curated structural models (2,637 models in 2,444 proteins), available at https://osf.io/bd2h4. Each variant is characterized by 106 sequence and structure features (see below). **VTS** comprises 5,901 human variants, 1,635 variants in other Eukaryotic proteins, 1,725 in Prokaryotic proteins, 122 Archael variants in proteins and 94 variants in viral proteins.

#### Acquiring structural models and homology models

We searched for crystal structures and homology models of proteins in **VTS** to maximize structural coverage. For proteins present in HumDiv without crystal structures in the PDB, we produced comparative models using Modeller (37,38). For proteins with sufficient variant annotation details in UniProt but without structures in the PDB, we extracted comparative models from ModBase (27) and SwissModel (36), selecting models with the largest sequence identity match to the query. All protein models were standardized to remove unwanted components (duplicate chains, ligands, metals and non-standard amino acids). This curation process resulted in 9,477 variants of 2,637 separate domains in 2,444 proteins (see Supplementary Figure S1).

### Protein variant characterization

Each protein variant is characterized by 106 features, 5 from sequence-based analysis, 17 from Rosetta ddg_monomer, 83 from Rosetta FastRelax and 1 additional feature generated using PROBE.

#### Sequence-based features from BLAST analysis

We find sequences similar to the query protein using PSIBLAST (2.2.25+, two iterations, pseudocount of two) (39) and extract five features directly from the output PSSM. At the protein position of interest, we use the PSSM log-likelihood of the native and variant amino acids (pssm_nat, pssm_mut) along with the position's information content (info_cont) as features. We also include an aminochange term that indicates broad chemical differences between the native and variant amino acid (see Supplementary Figure S2 and Supplementary Table S5).

#### Structure-based features from Rosetta analysis

**Figure 1.**
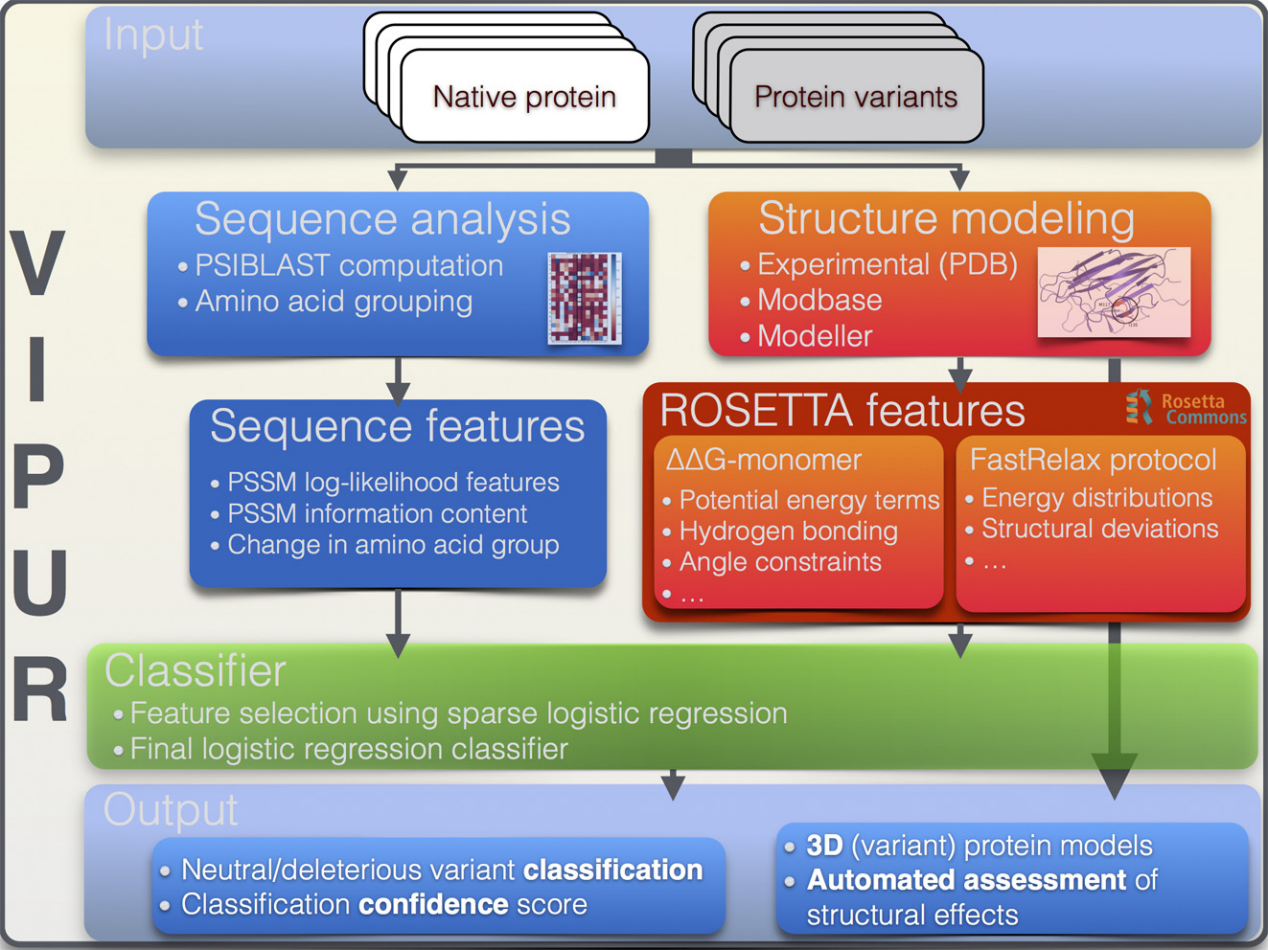
VIPUR analysis pipeline. Starting from a structural model of the native protein and a list of variants to be tested, VIPUR generates features using PSIBLAST and ROSETTA. Structure-based features are extracted from ROSETTA simulations comparing the native and variant protein structures. Variant structures are refined using the ddg_monomer protocol and the FastRelax protocol to consider a distribution of protein conformations. Features are combined in a logistic regression classifier that is trained on 9477 variants from over 360 species. VIPUR outputs the predicted label (deleterious or neutral), a confidence score, the top scoring 3D models of the variant protein structure and an automated interpretation of the variant effect derived from the weighted contributions of each feature to produce a physical description of protein disruption.

Stability differences between the native and variant protein structures are predicted by comparing their individual Rosetta Energy terms (25). The Rosetta Energy function combines physical and statistical potentials to approximate the energetic stability of protein structures and can be decomposed into individual scoring terms (26). We derive structure-based features from two different approaches for refining the local structure around the new amino acid; a fast approach approximating the change in Energy (Rosetta ddg_monomer (25), **17** features) and broader conformational sampling using Rosetta FastRelax (28,40) (**83** features). Both protocols (i) substitute the native residue for the variant amino acid, (ii) refine the variant structure, including protein backbone movements, to accommodate this change and (iii) compare the output structures using the Rosetta score terms (Figure 1, Supplementary Figure S2). To generate features for each variant, we follow Poultney *et al*. (23) and normalize structure-based features by comparing scores for a given variant to scores derived from Rosetta-relaxed ensembles of its native protein. We also include the accessible surface area at the position of variation as a feature, calculated using PROBE (41). Additional details on the methods of structural analysis and generation of the 106 features can be found in the Supplementary Material.

### Training a sparse logistic regression classifier

**Figure 2.**
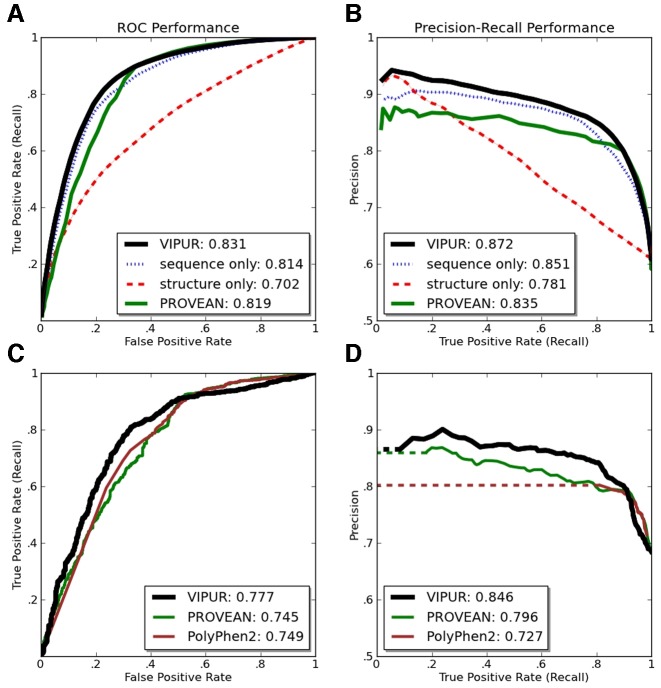
VIPUR training ROC and PR performance. ROC and PR curves for VIPUR and other popular methods. Curves (**A**,**B**) are averaged from 100 random splits (80% training, 20% testing) evaluated only on the leave-out testing sets. (A) Our combined classifier (black) has increased specificity compared to PROVEAN (green) with comparable sensitivity and higher AUROC than all other methods tested. PROVEAN and our sequence-only classifier have very similar AUROC but appear to emphasize sensitivity and specificity respectively. (B) VIPUR has notably increased AUPR over all other classifiers tested. Inclusion of the structure-based features improves classification (+2.5% accuracy) and dramatically improves ranking ability (.020 ΔAUPR). (**C**,**D**) We cannot directly compare performance of VIPUR to classifiers trained on the same variants (HumDiv) or restricted to predictions of human proteins. A VIPUR-like classifier was trained using 7935 variants from HumDiv and non-human proteins to compare performance with PolyPhen2 and PROVEAN on a set of 1542 human variants. The VIPUR-like classifier achieves higher AUROC (C) and AUPR (D) than both PolyPhen2 and PROVEAN.

VIPUR uses sparse logistic regression as a statistical classification framework to robustly discriminate between deleterious and neutral protein variants from the derived 106 sequence- and structure-based features and thus allows for a natural probabilistic interpretation of the outcome. Using stability-based feature selection, we identified a set of 20 non-redundant features that maximize the average generalization performance (42–44) of the logistic regression classifier (Supplementary Table S5 and Supplementary Figures S3 and 4). We evaluated the performance of this classifier on 100 independent random splits (80% training, 20% testing, split by proteins not variants) by means of average Receiver Operating Characteristic (ROC) and Precision-Recall (PR) curves (Figure 2, Supplementary Figure S6). Using the same strategy, we trained a sequence-only classifier using just the sequence-based features and a structure-only classifier using just the structure-based features (Supplementary Figure S4).

### Comparing VIPUR to other variant annotation methods

We compared VIPUR curves to several alternative methods, including the individual sequence-based and structure-based feature sets (Supplementary Figure S5), an optimized Support Vector Machine (SVM) with a radial basis function kernel (Supplementary Figure S9) and PROVEAN (Figure 2). Many popular variant annotation methods are only benchmarked on human variants, making interpretation of their predictions non-applicable for non-human variants, such as **VTS**.

We cannot properly compare performance between VIPUR and PolyPhen2 on the full **VTS** since it contains variants in non-human proteins and variants from PolyPhen2's training set (HumDiv). A set of 1,542 human variants included in **VTS** that are not included in HumDiv are used to compare a VIPUR-like classifier and PolyPhen2. To ensure a fair comparison, we retrained a VIPUR-like classifier (VIPUR*) on the remaining 7,935 variants of **VTS**. We calculated ROC and PR for VIPUR*, PolyPhen2 and PROVEAN on this set of 1,542 variants (Figure 2) and a subset of 383 variants found naturally in the human population (Supplementary Figure S7 and Supplementary Table S4). We also calculated ROC and PR curves of VIPUR to predictions from SIFT and CADD on 950 human variants with predictions available in dbNSFP (45) (Supplementary Figure S7). A set of 4,992 variants found in non-human species (included in **VTS**) are used to compare the performance of VIPUR, PolyPhen2 and PROVEAN on variants in other species (Supplementary Figure S8). To ensure this comparison does not overinflate any organism-specific contribution to performance, we calculated averaged ROC and PR curves on 100 random samples of 1000 variants, split by protein (from a set of 4,992 non-human variants).

### VIPUR software availability

VIPUR is currently available as an independent Python module requiring BLAST+, ROSETTA and PROBE (all freely available for academic use). Please see the VIPUR code for usage and analysis details, available at https://osf.io/bd2h4. The full predictions for all variants below, including structural models, are also available at https://osf.io/bd2h4.

### Classifying ClinVar annotated single nucleotide variant phenotypes

We demonstrate that VIPUR's deleterious predictions are an accurate indication of variant pathogenicity by classifying variants in the ClinVar database (29). ClinVar is a collection of human variants with annotated phenotypic effects, including variants with causative ‘pathogenic’ effects and ‘benign’ variants with no known disease effect. We expect VIPUR deleterious predictions to be enriched for variants with ClinVar ‘pathogenic’, ‘likely pathogenic’ or ‘risk factor’ annotations, termed pathogenic variants. We also expect VIPUR neutral predictions to be enriched for variants with ‘benign’ and ‘likely benign’ annotations, termed benign variants. Many variants in ClinVar contain variants with uncertain effects or conflicting annotations (e.g. ‘likely benign’ and ‘likely pathogenic’) including variants directly annotated with ‘uncertain significance’. We obtained models for 24,703 variants (in 4,016 proteins) in ClinVar from available structures in the PDB, ModBase and SwissModel out of 32,311 variants (in 7,188 proteins) that could be unambiguously matched to UniProt proteins. ClinVar contains many additional Single Nucleotide Polymorphisms (SNPs) entries that lack appropriate protein IDs, variant positions or annotations. Here we present predictions for VIPUR, PolyPhen2 and PROVEAN on 5,590 variants in ClinVar containing 498 benign variants, 1,797 pathogenic variants and 3,295 variants with uncertain annotation. Additional predictions for CADD, SIFT and PROVEAN were obtained from dbNSFP (45) to compare the score distributions between these methods.

### Obtaining inflammation disease-associated variants

To demonstrate detailed VIPUR predictions on disease-associated variants, we applied VIPUR to variants associated with inflammation diseases. We collected variants associated with various inflammation diseases and diabetes mellitus from entries in OMIM (46) and UniProt (35) by searching for the terms ‘Celiac disease’, ‘Crohn's disease’ and ‘diabetes mellitus’, and mapping these variants onto available protein structures. This resulted in 388 variants in 46 disease-associated proteins. We provide illustrative examples of different deleterious variants and functional sites (Figures 4 and 5 and Supplementary Figures S10 and S11).

### Classifying de novo mutations in the simons simplex collection

We tested VIPUR's ability to identify disease-associated variants by classifying *de novo* missense mutations in the SSC of sequenced exomes from families (quads and trios) with children having Autism Spectrum Disorders (ASD) (referred to as probands) (30–32) and unaffected siblings. Quad studies consist of exome sequencing for children with ASD, both of his or her parents and siblings with no intellectual disability or ASD phenotype. These studies identify *de novo* SNVs in children with ASD (variants not present in either parent) and examples of *de novo* variation from the unaffected siblings. For 2,814 mutations in the SSC, 2,226 mutations could be analyzed by all variant annotation methods tested (1,335 missense mutations found in proband children and 891 mutations in their unaffected siblings). For VIPUR, 1,644 mutations were mapped onto structures from the PDB, ModBase and SwissModel, considering models of all protein isoforms available for genes with alternative splicing. We predicted deleterious scores using VIPUR and applied our sequence-only classifier to the 582 mutations that could not be mapped to structure. For each mutation, we only considered the isoform prediction with the highest score, treating any deleterious prediction for a gene as indicative of deleteriousness. We compared the VIPUR, PolyPhen2 (HumDiv), CADD and SIFT predictions to the phenotype associated with each *de novo* mutation (proband or unaffected sibling) (45). These variant annotation methods are fundamentally trained to classify slightly different labels, however, all of these methods are used in practice to prioritize variants for further research and can be compared in their capacity to rank and segregate ASD-associated variants.

Since these methods have different scores, we consider the enrichment for proband mutations across score thresholds by calculating the ratio of proband to sibling mutations in different score bins. Although these classification methods differ, we expect high scores (deleterious, ‘damaging’, ‘intolerant’) to be enriched for proband mutations and low scores (neutral, ‘non-damaging’, ‘tolerant’) to be enriched for mutations found in unaffected siblings (Figure 6). We consider the correlation between this enrichment ratio and each output score across score thresholds and also the enrichment ratios found at the score cutoff of .5. We verified that this method of comparison is robust to the number of bins (Supplementary Figure S14) and the score threshold used (Supplementary Figure S15).

## RESULTS

### Accuracy and generalization of the VIPUR classifier

Combining sequence-based features and structure-based features enables VIPUR to accurately and precisely identify deleterious variants, achieving >90% precision on the highest scoring 40% (over 3,800 variants above score cutoff of .7, Figure 2B). VIPUR achieves a higher AUROC and AUPR than PROVEAN and other methods tested (Figure 2). Scores that clearly indicate confident predictions are essential for prioritizing variants and deleterious proteins. Filtering predictions with our confidence score raises the accuracy from 81% with no ranking (scores above .5 are considered deleterious) to >94% accuracy for scores above .95. We tested both the classification (in-set) and generalization (out-of-set) performance of VIPUR and report here only the generalization performance (Figure 2) since this is characteristic of VIPUR's behaviour on new variants. The classification and generalization performance converge as the training set size increases demonstrating that VIPUR predictions are robust and the classifier is not overfit to the training set (Supplementary Figure S4). Classifiers trained on only the sequence-based features correctly predict 78% of the dataset, providing a high baseline performance, while the structure-based features cause VIPUR output scores to scale with precision, indicating a clear estimate of prediction confidence. Adding structure-based features improves performance by recovering improperly classified neutral samples with a slight change in deleterious sensitivity, suggesting these features help identify misclassifications made by the sequence-based features (Figure 2A).

### Comparison to other classifiers

We compare performance of our combined classifier to PROVEAN, PolyPhen2 and multiple classifiers trained on our own features (structure and sequence features only). PROVEAN is a popular variant annotation method that extends the SIFT framework for identifying deleterious variants. We compare performance on the entire **VTS** to PROVEAN since it can interpret variants in any organism without additional training and is not overfit to any particular training set. Using the full **VTS** our combined classifier performs better than PROVEAN with improved classification (AUROC 0.831 over PROVEAN's 0.819) and notably improved ranking ability, quantified by our AUPR of 0.872 over PROVEAN's 0.835; over 20% of the AUPR not covered by PROVEAN (Figure 2A). Our sequence-only classifier displays similar performance to PROVEAN, with nearly identical AUROC (Figure 2). The ‘flat’ shape of the PR curves for sequence-based classifiers may be a general property of these feature sets, providing generalized predictions without clear specificity since they do not identify any specific mechanism of protein disruption. These similarities also suggest that our sequence-based features appropriately capture the deleterious signal within multiple sequence alignments (when used with logistic regression).

We are unable to consistently compare performance of VIPUR to popular human-specific methods on the full **VTS**. For example, PolyPhen2 does not officially support prediction on non-human variants and is trained on HumDiv (contained in **VTS**). Accordingly, we compare our method to PolyPhen2 over a subset of 1,542 human variants in **VTS** using a classifier similar to VIPUR but trained on the remaining 7,935 variants of **VTS**, termed VIPUR*. VIPUR* produces ROC curves similar to PROVEAN and PolyPhen2 with notably improved AUPR on this set of human variants (Figure 2C and D). PROVEAN and PolyPhen2 perform very similarly on these variants although PolyPhen2 predictions are restricted to a small region of the PR landscape (PolyPhen2 scores are highly degenerate, a large number of predictions obtain a score of ‘1’). The decrease in performance for VIPUR and PROVEAN on this set of variants suggests these variants represent mutations that are different from the rest of **VTS**. VIPUR* appears overfit, due to the lack of diverse neutral annotations during training (HumDiv neutrals are all pseudomutations) and we included all available variants with neutral annotations when training the combined classifier to eliminate this overfitting. We also contrast the performance of our logistic regression classifier with a SVM classifier using an optimized Radial Basis-Function kernel (Supplementary Figure S6). Our logistic regression classifier achieves higher accuracy, AUPR and AUROC than the SVM classifier with fewer features (reduced complexity), superior generalization and direct interpretability (Supplementary Figure S6B).

We compared performance of VIPUR to PolyPhen2, PROVEAN, SIFT and CADD on 950 human variants with predictions available in dbNSFP. VIPUR achieves higher AUROC and notably higher AUPR than all of the other methods tested (Supplementary Figure S7). PolyPhen2, PROVEAN, SIFT and CADD all have very similar ROC curves on this dataset including an increased sensitivity for deleterious variants in lower confidence scores. CADD demonstrates a better ranking ability (AUPR) than PolyPhen2, PROVEAN and SIFT on this dataset.

PolyPhen2 does not officially support predictions on variants found in species other than human, however, it is commonly used to assess deleterious effects since it can run on any input sequence. To assess non-human performance, we compared VIPUR predictions to PolyPhen2 and PROVEAN on a set of 4,992 non-human variants using averaged ROC and PR curves from 100 random samples of 1000 variants to reduce any organism-specific effect on performance (Supplementary Figure S8). Predictions on these non-human variants achieve similar performance to the 1,542 human variants dataset for all three methods. VIPUR achieves higher AUROC and notably higher AUPR than the other methods. PolyPhen2 and PROVEAN perform very well even though they are not explicitly trained on variants in non-human species. As with predictions on other datasets, PolyPhen2 predicts a large number of variants to have high-confidence deleterious scores, inhibiting PR performance.

Across these comparisons, VIPUR consistently achieves higher AUROC and often has a dramatically higher AUPR. Structure-based features provide this improved ranking ability by identifying variants with deleterious sequence-based feature scores that do not appear to energetically destabilize the protein. This improved ranking allows VIPUR to reduce large sets of unlabelled variants to small sets of high-confidence predictions that are enriched for true deleterious effects.

We investigated prediction trends of VIPUR across numerous protein properties including the source of data, species of origin, fold classification, functional annotation and model quality (using Pearson chi-squared test, see Supplementary Material). These trends show a slightly increased false negative rate for eukaryotic proteins and a slightly increased false positive rate for prokaryotic proteins. This is likely caused by simple label imbalance since the majority of neutral-labeled variations are in eukaryotic proteins. While VIPUR generalizes very well across diverse protein functions, the structure-only classifier has an increased false negative error rate on several DNA- and RNA-associated proteins, suggesting that simulating these interactions will improve the accuracy of our structural modelling (DNA and RNA are absent in our structural models). We have verified that VIPUR's performance is the same for proteins with many variants in **VTS** and proteins with no other variants in the training set. This demonstrates that VIPUR is not overfit to specific sequence/fold properties, a confounding form of overfitting (47) (Supplementary Table S3).

### VIPUR predictions match ClinVar phenotypes

We tested VIPUR's capability to distinguish pathogenic variants from benign variants by classifying SNVs in the ClinVar database. ClinVar's curated annotations include benign variants with no known effect on disease and a large collection of pathogenic variants with various causal roles in genetic disorders and disease susceptibility. The variant annotations in ClinVar do not directly match VIPUR labels, but we expect ClinVar pathogenic variants to be enriched for deleterious VIPUR predictions and for ClinVar benign variants to be enriched for neutral VIPUR predictions. We emphasize that not all pathogenic variants are deleterious and many deleterious variants appear benign when they do not have clear biological phenotypes.

**Figure 3.**
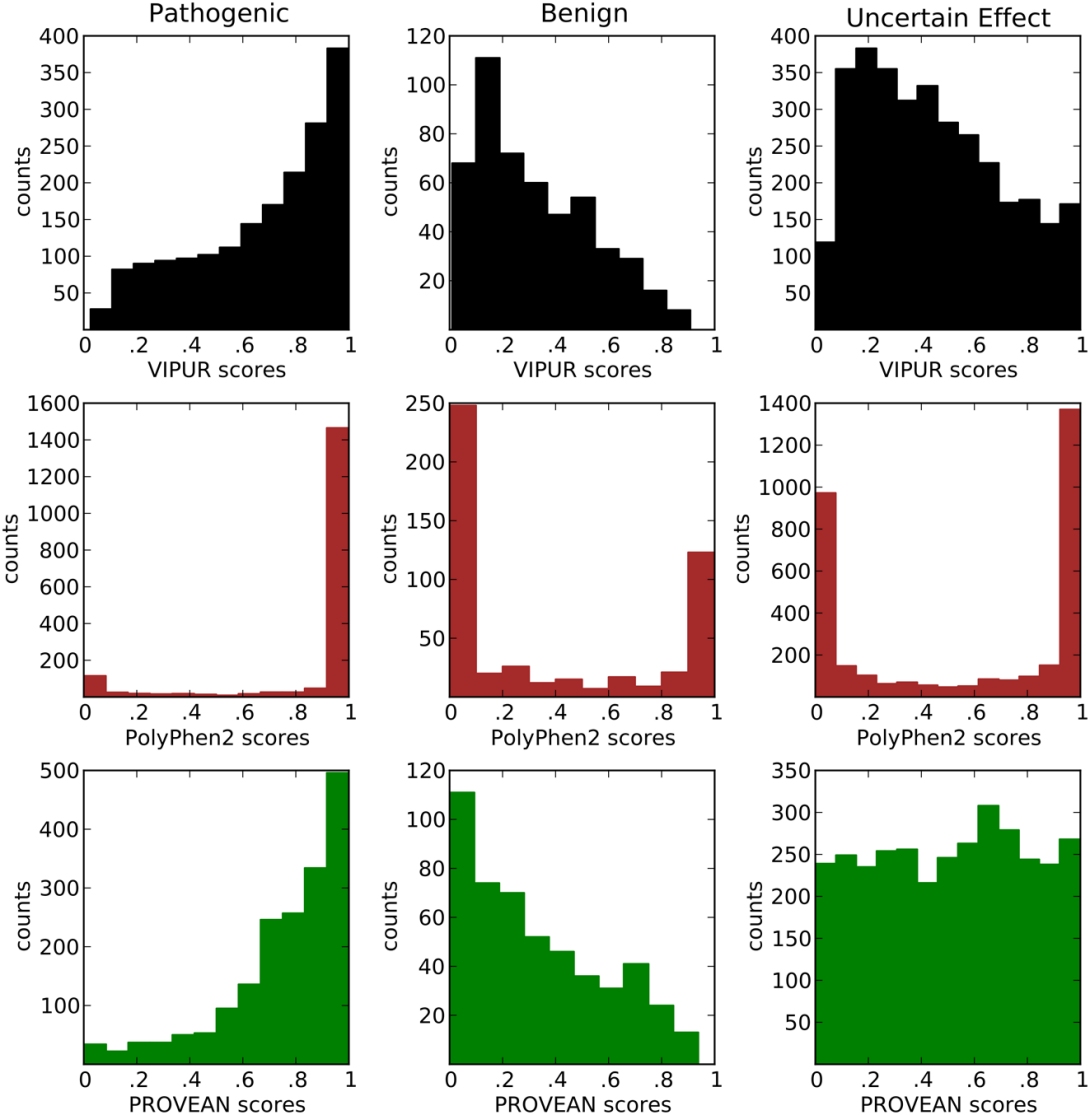
VIPUR scores clearly identify pathogenic variants. VIPUR predictions on ClinVar variants match expectations from their phenotype annotations. Left: Pathogenic variants have a skewed distribution of VIPUR deleterious scores (>.5) and have similar trends for PolyPhen2 and PROVEAN. Center: Benign variants have a broad distribution of VIPUR neutral scores (<.5), while PolyPhen2 pushes variants to high and low scores. We expect most genetic variations to be benign and unlikely to disrupt protein function, however, datasets like ClinVar have a high pathogenic label bias. Right: Predictions on ClinVar variants annotated with uncertain effect highlight VIPUR's ability to identify a small set of likely deleterious variants, while PolyPhen2's high false positive rate leads to an overwhelming number of high-confidence ‘probably damaging’ predictions. VIPUR's score distribution resembles the benign variants with a small set of confident deleterious predictions while PROVEAN scores are uniformally distributed.

Pathogenic variants have a highly skewed distribution of VIPUR deleterious scores, while benign variants have a broad distribution of neutral scores (Figure 3). PolyPhen2 scores tend towards high and low values that also clearly distinguish between pathogenic and benign variants. PROVEAN scores are distributed similarly to VIPUR scores, matching our expectations for ClinVar variants. All three methods are designed to highlight deleterious variants and must be able to clearly identify variants with strong evidence of deleteriousness. In this comparison, VIPUR has a higher specificity than PolyPhen2 with a reduced sensitivity. ClinVar itself has a high label bias with a 7:2 proportion of pathogenic:benign annotations. Training on datasets with a large label imbalance can inherently offset the sensitivity/specificity tradeoff of a classifier and must be avoided by training on samples that accurately represent the category labels. Prediction methods of other variant annotation methods resemble VIPUR predictions and match our expectations for score distributions on pathogenic and benign variants (Supplementary Figure S12).

Predictions on ClinVar variants annotated as uncertain effect demonstrate the differences in error rates between these methods (Figure 3, Supplementary Figure S7). VIPUR predictions are predominately neutral with a small set (208/3,295, 6%) of highly confident deleterious predictions, while PolyPhen2 predicts over seven times as many ‘high-confidence’ pathogenic variants (1,435/3,295, 44%!). PolyPhen2 is explicitly designed to identify variants associated with complex traits (13) and could simply be identifying variants that are ‘probably damaging’ in other disease susceptibility traits. Even if this is the case, this high positive prediction rate reduces PolyPhen2's ability to identify small sets of highly confident predictions for subsequent investigation.

PROVEAN predictions are nearly uniform without enrichment at the highest and lowest scores or a score distribution resembling either benign or pathogenic variants. Without reliable labels for ClinVar variants of uncertain effect, the accuracy of these predictions cannot be evaluated. VIPUR is the only method tested with a score distribution for these variants resembling its benign variant distribution and places the fewest number of these variants into the highest confidence bins (Supplementary Figure S12 and Supplementary Table S6). Nearly all of these methods identify some aspect of deleteriousness although classification of variants with uncertain labels is very diverse between these methods. Several variant annotation methods may have artificially high false positive rates and comparisons between these methods will obtain similar score distributions when benchmarked on datasets with a large deleterious label bias (like ClinVar). The uncertain effect variants likely have a different label ratio, leading to the diverse behaviour of these methods.

### Examples of detailed structural annotation for deleterious variants associated with human diseases

We demonstrate VIPUR's applications by predicting deleterious variants among a small set of inflammation- and diabetes-associated variants. Genome Wise Association Studies and exome sequence studies of disease conditions reveal many candidate variants and genes by associating variants to traits and conditions. Some of these variants will be deleterious and may have large effects on the disease phenotype. The variants collected here do not necessarily have causal roles in inflammation or diabetes, unlike the ClinVar pathogenic variants which have established effects, but instead provide examples of VIPUR prioritization and interpretation. We collected proteins and variants associated with the terms ‘Celiac disease’, ‘Crohn's disease’ and ‘diabetes mellitus’ from OMIM (46) and UniProt (35), identifying 388 variants in 46 disease-associated proteins (in 102 models). We predicted VIPUR scores for each variant and interpreted the structure-based features of each variant model. Predictions on the entire set of disease-associated variants are available at https://osf.io/bd2h4.

Out of 388 variants, we predict 205 are deleterious with 108 having confidence scores above .8. UniProt annotations for these deleterious variants have several keywords describing damaging effects. These descriptions, however, do not meet our curation standard for a deleterious label in **VTS** but are suggestive of the variant's functional impact. Our physically intuitive structure-based features allow VIPUR to automatically produce structural hypotheses about the physical causes of deleteriousness. We include a summary of the structure-based features that contribute to the deleterious classification with each prediction, indicating disrupted hydrogen bonds, disulfide bridges, improper packing and other structural defects. Many deleterious variants destabilize the protein native state by introducing a steric clash or otherwise preventing proper packing arrangements. In this dataset, variants in NR3C1, HNF1A, NEUROD1 and SIAE all clearly disrupt packing interactions. During classification, features like the Rosetta van der Waals repulsive term (fa_rep) contribute a large deleterious score, allowing automated identification of packing disruption. While these amino acid changes dramatically alter the side-chain shape and size, amino acid side-chain interactions are most easily identified using 3D contacts in the protein structure. VIPUR's structure-based features automatically detect disrupted side-chain interactions using Rosetta's statistical potentials. In this dataset, variants in LEP, AKT2 and TGM2 are predicted to disrupt specific interactions that stabilize the folded protein. These examples are representative of automated VIPUR interpretations but many long-range effects require sampling protein backbone conformations to properly interpret variant effects.

**Figure 4.**
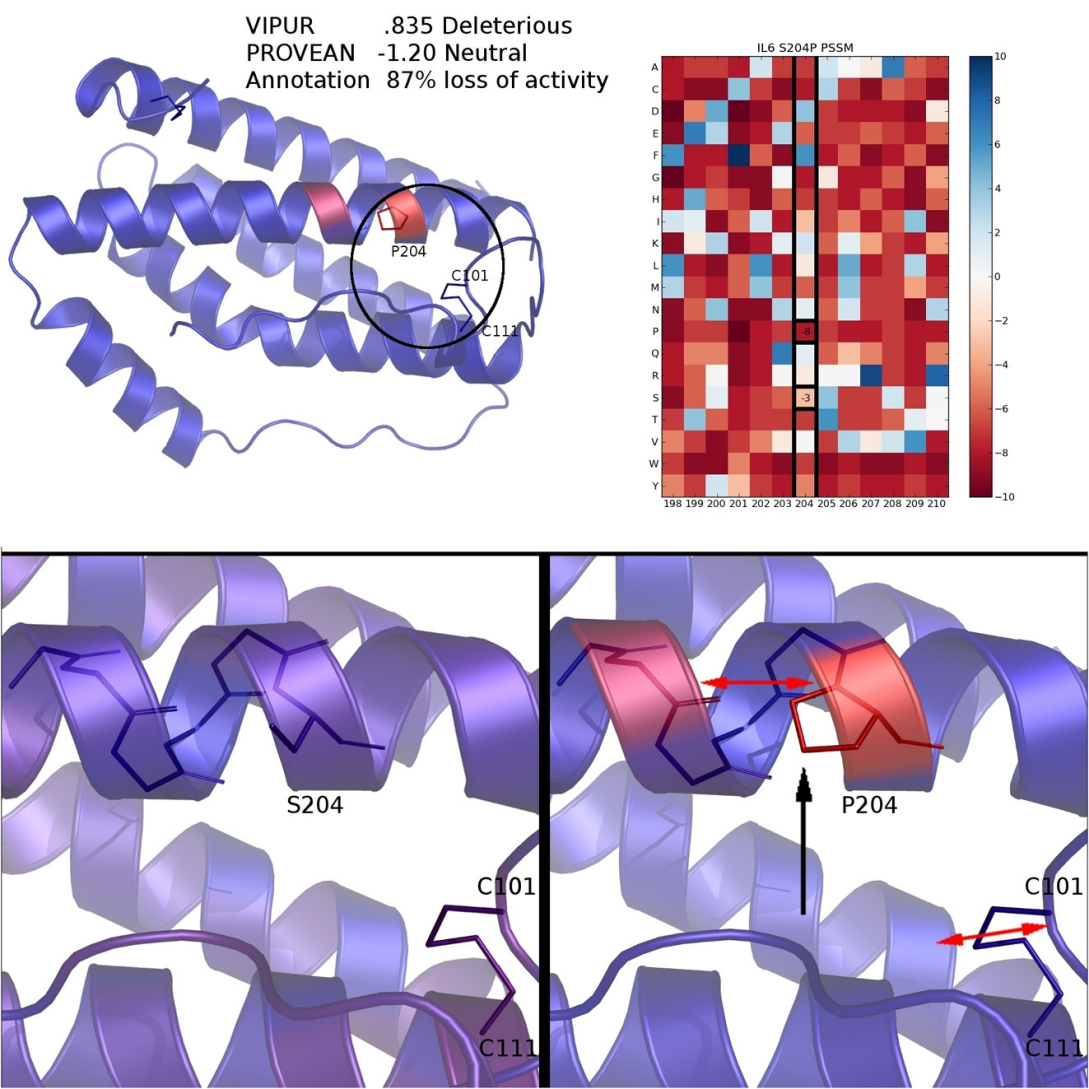
S204P disrupts a critical helix interface in IL6. VIPUR predicts S204P is deleterious (.835), matching the UniProt annotation ‘87% loss of function’ and infers the deleterious label due to destabilized disulfide bond, while PROVEAN predicts S204P is neutral (−1.20 score). Every residue in IL6 is coloured by the difference in Rosetta energy between the native and variant protein structures, highlighting the destabilization introduced by S204P (top left). The PSSM generated by PSIBLAST does not indicate strong conservation for serine at position 204 (top right, PSSM columns shown for surrounding residues). The native S204 structure has a stable interface (bottom left, residues coloured by Rosetta energy of a representative model) but becomes destabilized in the P204 variant model (bottom right). Perturbing this helix could accommodate the proline destabilization, however, this strains the nearby C101–C111 disulfide bond (bottom right), leading to an accurate deleterious prediction.

Many physical interactions within a protein are far apart in sequence, limiting the insight provided by methods that assume protein positions are independent. VIPUR can correctly identify mutations that disrupt these interactions by analyzing a 3D structural model of the protein, even when destabilization occurs far from the mutated position. We identified several cases where mutations disrupted interactions between elements of secondary structure, a deleterious effect captured by VIPUR but missed by sequence-based methods. The S204P variant of IL6 is associated with numerous inflammation diseases (Figure 4) and annotated in UniProt as ‘87% loss of activity’. While PROVEAN predicts this variant is neutral (−1.20 score), VIPUR predicts this variant is deleterious with high-confidence (.835) and infers that it disrupts a disulfide bond. Position 204 is not close enough to destabilize the nearest disulfide bond, C101–C111, by direct interaction (Figure 4, bottom), however, conformational rearrangements that accommodate P204 disrupt the interface between helix four and helix seven, straining this disulfide bond. These subtle structural changes cannot be detected with a multiple-sequence alignment or structural modelling of a single conformation. V117M of ADIPOQ also appears neutral in a PSSM and PROVEAN (−2.00 score), but interactions between protein backbones with β-strand pairing inform a deleterious prediction by VIPUR (Supplementary Figure S10). V117 is physically close to I135 on an adjacent β-strand and mutation of V117 to methionine introduces a clash between these positions that cannot be accommodated without breaking inter-strand hydrogen bonds, destabilizing the β-sheet (Supplementary Figure S10, bottom right). These examples demonstrate the clarity and scope of structural modelling to detect destabilizing mutations, highlighting the limited performance of sequence-based methods at positions without strong conservation.

**Figure 5.**
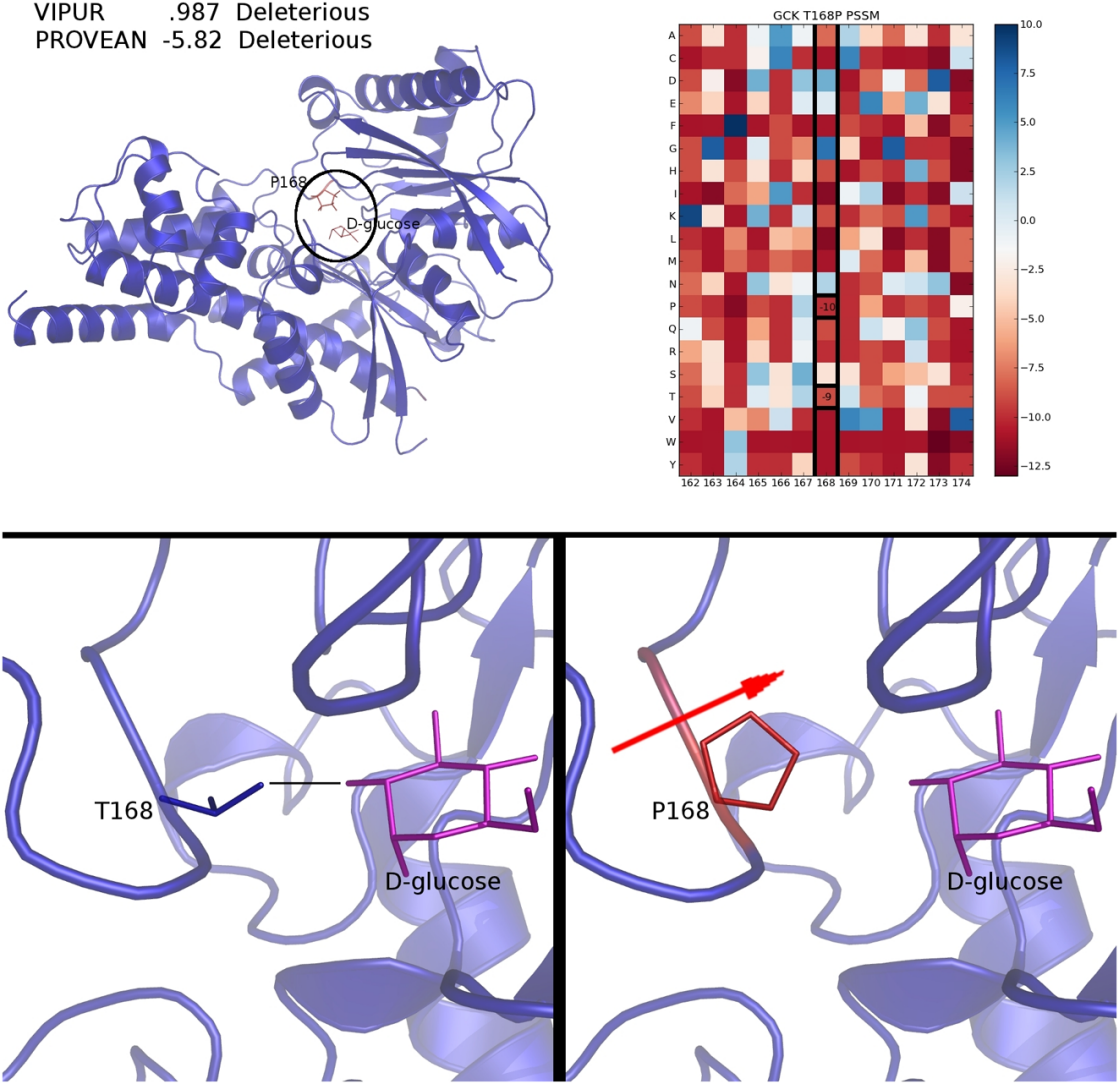
T168P destabilizes an active site loop in GCK. T168P is predicted deleterious (.987) due to disrupted backbone interaction (hydrogen-bonding) and is also predicted deleterious confidently by PROVEAN (−5.82). Every residue in GCK is coloured by the difference in Rosetta energy between the native and variant protein structures, highlighting the high energy of P168 (top left). The PSSM generated by PSIBLAST indicates both the native and variant amino acids are not favoured at position 168 (top right, PSSM columns shown for surrounding residues). The native T168 forms a hydrogen bond to the substrate, D-glucose (bottom left, ligand position from PDB 3F9M, residues coloured by Rosetta energy of a representative model), which is absent in the P168 variant. Although interaction with D-glucose is not simulated during classification, VIPUR predicts proline is destabilizing due to disrupted backbone hydrogen bonding, suggesting other active sites can be accurately classified even without bound ligands.

Beyond long-range interactions, VIPUR can also detect destabilization at active sites and binding interfaces. The protein Glucokinase, GCK, has many diabetes-associated variants, including several high-confidence predictions in this dataset: T168P, G299R, W257R and G385V. Position 168 is a conserved glycine in the PSSM and predicts both the native threonine and variant proline are similarly unfavourable. This conservation causes PROVEAN to predict T168P as deleterious (−5.82) even when the native threonine is just as disfavourable as the variant (based on sequence analysis), yet known to make a hydrogen bond with the substrate D-glucose (from PDB 3F9M). Our structural model does not include this interaction with D-glucose (all ligands are removed) yet VIPUR still predicts mutation to proline is highly destabilizing (.987) based on the unfavourable backbone conformation of proline (Figure 5) at this *structurally conserved* binding site. We observe a similar pattern at other ligand and metal binding sites, such as ZFP57 H374D (not shown), where structure-based features produce confident deleterious predictions even without explicitly including the ligand or metal in the structural model. This suggests that interaction sites have conserved structural properties that can help identify deleterious variants and that VIPUR predictions may identify disrupted active sites even when the substrate is unknown.

### Confident prediction of deleterious de novo mutations associated with autism spectrum disorders

To demonstrate VIPUR's ability to prioritize disease-associated genetic variants in the absence of curated labels, we ran VIPUR on the SSC. While VIPUR is trained to predict disruption of protein function, many other variant annotation methods are trained to predict different specific labels. Despite differences in training and intended application, all of these methods are practically applied to rank probable disease-associated candidate variants from large sets of SNVs and can be compared to each other for this task.

The SSC is a set of *de novo* SNVs where the genotypes of children with ASD are compared to their parents, identifying *de novo* variation. These quad studies require genomic comparison to both parents, the child with ASD and an unaffected sibling to provide samples of *de novo* variation found in children without ASD. Many of the variants in the SSC may be *non-causal* for ASD or otherwise contribute weak effects to complex behavioural phenotypes, obscuring the deleteriousness and pathogenicity of these variants. We expect the deleterious/damaging/intolerant predictions from these classification methods to be enriched for *de novo* mutations found in children with ASD (probands) while neutral/no effect/tolerant predictions should be enriched for variants in unaffected siblings.

**Table 1.** Predictions on the Simons Simplex Collection

2226 *de novo* variants from the SSC (1335 proband, 891 sibling, 1.50 label bias)					
Method	BLOSUM62	**VIPUR**	SIFT	PolyPhen2	CADD
proband-D	907	554	779	769	747
proband-N	428	781	556	566	588
sibling-D	566	348	499	494	499
sibling-N	325	543	392	397	392
#>.95	0	43	545	607	186
proband enrich	1.60	1.59	1.56	1.56	1.50
sibling enrich	1.32	1.44	1.42	1.43	1.50
Spearman	0.90	0.87	0.54	0.53	0.13
Spearman *P*-value	0.08	**2.68e-3**	0.11	0.12	0.73
Pearson	0.92	0.86	0.44	0.49	0.03
Pearson *P*-value	**0.03**	**1.39e-3**	0.20	0.15	0.93

proband-D: proband mutations in deleterious predictions (true positives) at .5 cutoff, proband-N: proband mutations in neutral predictions (false negatives) at .5 cutoff, sibling-D: sibling mutations in deleterious predictions (false positives) at .5 cutoff, sibling-N: sibling mutations in neutral predictions (true negatives) at .5 cutoff, proband enrich: the ratio of proband-D/sibling-D, sibling enrich: the ratio of proband-N/sibling-N. *P*-values in **bold** are less than the **0.05** significance threshold.

These methods all output confidence scores that are scaled from 0 to 1 with high scores predicting deleterious effects and low scores predicting neutral effects. When thresholding prediction scores at .5, all methods tested have a higher proportion of proband mutations in deleterious predictions and a lower proportion in neutral predictions, however, none of the methods appear notably enriched. Since the classification threshold is arbitrary, no single threshold will be appropriate for all methods, however, we expect proband enrichment to be proportional to the confidence score. We count the number of proband and sibling mutations found in each score bin and compare this ratio to the confidence score of that bin. We calculate the correlation between the annotation confidence score and proband enrichment to compare each method's ability to confidently identify disease-associated mutations.

**Figure 6.**
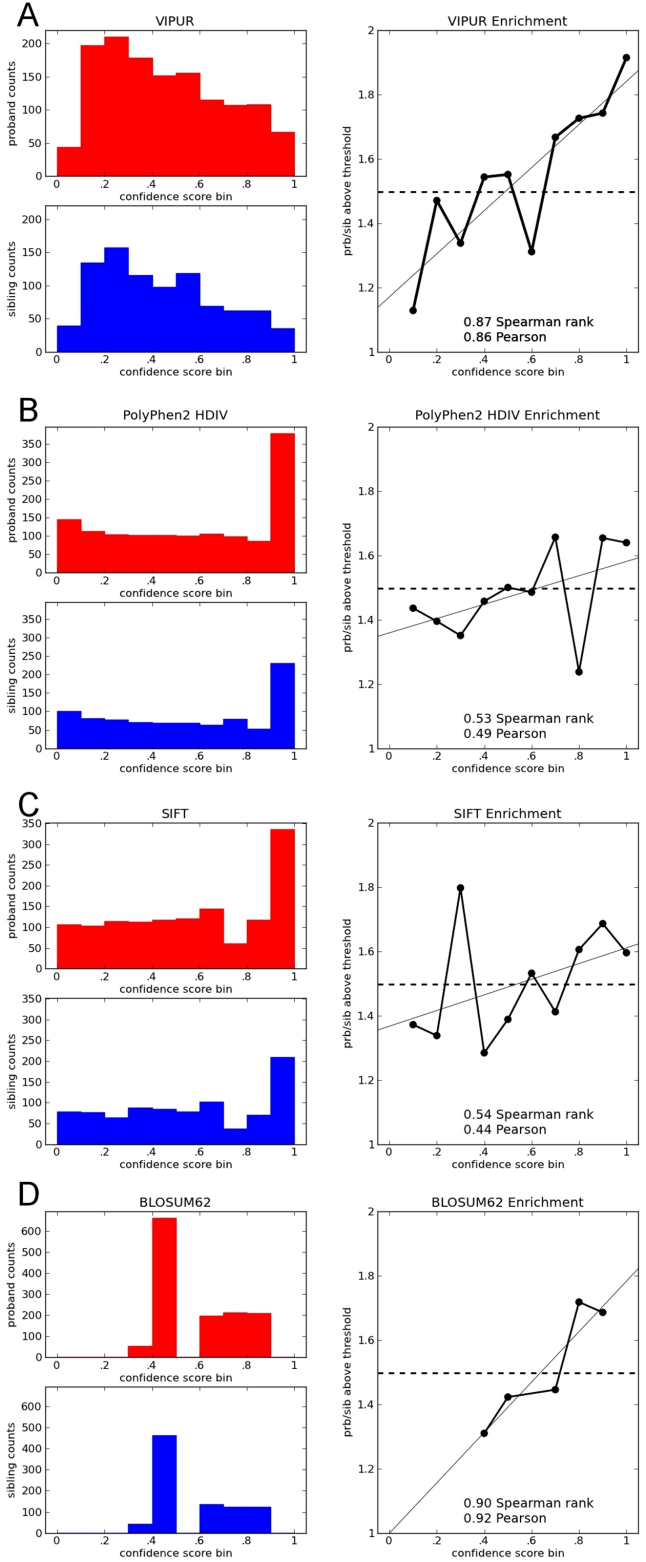
VIPUR deleterious predictions are enriched for autism-associated mutations. Predictions for various methods on the SSC, containing 1,335 *de novo* mutations found in children with autism-spectrum disorders (probands) and 891 *de novo* mutations found in unaffected siblings. For each prediction method, the distribution of confidence scores is shown for proband (red) and sibling (blue) mutations. The ratio of these counts for each score bin is shown along with the background expectation (dashed line, 1.50, 1,335/891). We expect high deleterious/damaging scores to be enriched for proband mutations and low scores to be enriched for mutations found in siblings. (**A**) VIPUR predicts most mutations in both sets have neutral effects while properly enriching high deleterious scores for proband mutations and de-enriching low scores for probands mutations. (**B**) PolyPhen2 effectively splits mutations into a high-confidence bin versus everything else, however, this top scoring bin is not strongly enriched for proband mutations. (**C**) SIFT scores are distributed similarly to PolyPhen2 with similar overall correlation, however, its fluctuations around the background expectation are different. (**D**) Using the simple BLOSUM62 score (negative scores are deleterious) yields an excellent enrichment for proband mutations, however, the scores are not truly continuous leading to fewer scores (smaller *P*-value).

The simple BLOSUM62 matrix achieves an impressive enrichment for proband mutations despite having only seven distinct values for mutations in this dataset. Surprisingly, PolyPhen2, SIFT and CADD do not display significant enrichment across score thresholds, although SIFT and PolyPhen2 have trends in the proper direction for intolerant/damaging predictions (Figure 6, Table 1, Supplementary Figure S13). VIPUR is the only method to obtain significant Spearman (rank) and Pearson correlations across score thresholds, properly enriching deleterious predictions for proband mutations and removing proband mutations from neutral predictions. VIPUR predictions also fit our intuition that the majority of variants in this dataset are predicted to have a neutral effect on protein function. PolyPhen2 is explicitly designed to predict ‘rare alleles at loci potentially involved in complex phenotypes’ (13) and may be accurately identifying variants associated with non-specific complex phenotypes in probands and siblings. However, this would suggest that there is only an extremely slight difference in the expected load of deleterious mutations. Any difference between these methods due to score thresholds or the categorical nature of these predictions, as opposed to their continuous output metrics, is eliminated when predictions are accumulated across score thresholds where VIPUR maintains a higher enrichment (Supplementary Figure S15).

Many of these variant effect annotation methods are trained and/or benchmarked on datasets with a high label bias. This label imbalance likely contributes to the inflated positive rate we observe for many methods tested here (Supplementary Figure S7). Since we are primarily concerned with the efficient identification of candidates for follow-up studies, proper *ranking* of pathogenic variants is essential for highlighting causal mutations and can be severely confounded by high positive rates for *de novo* mutations. At the confidence score threshold of .95, VIPUR predicts 43 variants are very likely to have disrupted molecular functions which may contribute to ASD, while PolyPhen2 predicts 607 variants with high confidence. While these confidence thresholds are arbitrary, we verified that this trend is invariant to the number of bins used (Supplementary Figure S14) or the classification thresholds used (Supplementary Figure S15).

## DISCUSSION

VIPUR is a variant annotation method that is designed to identify deleterious variants by analyzing conservation and protein structural energetics. The VIPUR deleterious and neutral labels are learned from curated annotations of variants with clear effects on protein molecular functions and are not restricted to variants with known pathogenicity for any particular disease or any single organism. VIPUR has superior performance to PROVEAN and PolyPhen2 on out-of-set evaluations drawn from **VTS**. Our structure-based features enhance the ranking ability of VIPUR, leading to an improved precision for variants with higher deleterious scores. We demonstrate that VIPUR-predicted labels match expectations for the pathogenic and benign phenotype annotations in the ClinVar database. All other variant annotation methods tested also match these expectations as well, although the methods notably disagree about ClinVar variants with uncertain effect. Examples of VIPUR predictions on inflammation- and diabetes-associated variants demonstrate the clarity of structure-based features to explain the specific causes of protein deleteriousness. These automated structural interpretations are only possible using structure refinement techniques that can identify long-range structural disruption. Predictions on the SSC show that VIPUR deleterious predictions are more enriched for *de novo* mutations found in children with ASD than any other method tested. While **VTS** and ClinVar have a strong deleterious label bias, we expect most genetic variations to have neutral effects and VIPUR consistently predicts neutral scores for collections of variants of unknown significance.

Our current method allows us to accurately predict and interpret many protein variants, however, several substantial improvements to this method are on the horizon. Successful prediction of variants in IL6 (Figure 4) and ADIPOQ (Supplementary Figure S10) demonstrate that VIPUR can accurately predict the effects of amino acid substitutions even when disruption occurs at a distant region of the protein structure. This suggests that VIPUR could predict the functional effects of multiple mutations within the same protein, even though these variants are not currently included in **VTS**. Thus far **VTS** includes 323 of the 400 possible single amino acid transitions. Although we observe nearly unbiased predictions across these amino acid transitions, VIPUR has slightly reduced performance for some substitutions with changes in polarity (Supplementary Table S2). More advanced electrostatics modelling in the context of our predicted structure ensembles will likely improve classification for these transitions (48). In addition to more sophisticated electrostatic features, many additional features are likely to improve performance, such as individual amino acid properties. Recent improvements to the Rosetta framework make it possible to incorporate DNA, RNA, metals and other cofactors into our structural models which will further improve our structure-based features and interpretation. Improved Rosetta protocols for modelling membrane environments, including transmembrane-specific conformational sampling and a membrane energy function with depth-dependent solvation and hydrogen bonding terms, will expand our coverage to include variants in transmembrane environments (49,50).

**VTS** currently includes 9,477 annotated variants in more than 360 species with 106 features for each variant and structural models from the PDB and homology models. Independent of VIPUR, this dataset is a valuable resource for researchers in computational biology and machine learning communities to develop and test novel classification methods. We are currently expanding **VTS** to include annotated variants with multiple substitutions, nearly neutral variations, variants in transmembrane proteins (50), alternative comparative models using multi-template homology modelling (38) and known binding interactions including variants at DNA- and RNA-protein interfaces. These advances will make VIPUR applicable to an even wider range of protein variants, further contributing to our understanding of structure-function relationships. Given the relatively distinct chemical environments and conformational motions between intrinsically disordered protein regions, transmembrane proteins and traditional cytosolic proteins, we expect individual classifiers trained for each type of protein region will perform better than a marginal classifier trained on all types combined. While the PDB does not include models of all proteins, human proteins are abundant and available models in ModBase and SwissModel help increase the structural coverage. Of the 32,311 protein coding variants in ClinVar (in 7,188 proteins) that could be unambiguously matched to proteins in UniProt, 24,703 (in 4,016 proteins) had structures available in either the PDB, ModBase or SwissModel (76% of variants covered, 55% of proteins). We apply our sequence-only classifier to protein variants lacking structural models and will continue to improve this rapid classification method. Although structural coverage limits our ability to classify all protein variants, VIPUR still identifies candidate genes and causal variants within large genomic datasets, highlighting only the variants with structural evidence of large effects.

## CONCLUSION

VIPUR has been designed to identify and interpret deleterious protein variants across multiple species and sources of variation. To achieve this generalization, we have collected and curated **VTS**, a dataset of protein variants with annotated functional and physical effects on protein molecules. VIPUR is currently the only method that predicts loss of molecular function directly, rather than ‘pathogenicity’, ‘damaging’ or some related label. While the differing labels of variant annotation methods can complicate comparisons, all of these methods are used to rank and prioritize variants for future investigation. VIPUR's superior classification performance and ranking stem from a seamless integration of high-quality sequence and structure information (Figure 2) and Rosetta's ability to find low energy backbone conformations that can accommodate neutral substitutions and indicate long-range disruption of deleterious substitutions. Unlike other methods, VIPUR uses automated structural analysis to make a detailed 3D model of each variant and subsequently infer the physical origin of deleterious predictions, generating hypotheses and interpretations previously achievable only by tedious manual inference.

We have demonstrated that VIPUR predictions are informed by protein structural constraints that cannot be identified using a multiple sequence alignment or a static protein structure alone (Figures 4 and 5). VIPUR can automatically highlight protein variants involved in human diseases that disrupt protein function and is applicable to nonsynonymous SNVs in proteins with reliable structural models. Although VIPUR predicts variants with disruption of biophysical function, this label matches expectations of biological phenotypes and predicts fewer false positives than many current variant annotation methods, a problem confounded by the incoherence of label bias between traditional benchmarks (more pathogenic examples than neutral examples) and real applications (where most single variants are expected to be neutral). While other methods lack the specificity required to identify neutral variation, VIPUR can clearly distinguish deleterious variants from neutral variants (Figure 6A and B). Previous advances in deleterious variant prediction have often focused on improving recall and global accuracy but failed to explain the origin of deleterious variation. Here, we demonstrate how these pathogenicity detection methods are great tools for initially filtering and identifying potential causal variants, however additional analysis, such as structural model analysis, is required to further refine candidates. VIPUR can identify deleterious protein variants and provide structural explanations for disrupted protein function. We hope that VIPUR will contribute to our understanding of structure-function relationships, particularly for the interpretation of *de novo* mutations and disease-associated variants.

## Supplementary Material

SUPPLEMENTARY DATA
