# Supplementary material for: Robust classification of protein variation using structural modelling and large-scale data integration
Source: Nucleic Acids Res. 2016 Feb 28;44(6):2501–13. doi: 10.1093/nar/gkw120 (PMC4824117; doi:10.1093/nar/gkw120)
Supplement: SUPPLEMENTARY DATA [file supp_44_6_2501__index.html]

Robust classification of protein variation using structural modelling and large-scale data integration — SUPPLEMENTARY DATA 

# Robust classification of protein variation using structural modelling and large-scale data integration

## SUPPLEMENTARY DATA

- SUPPLEMENTARY DATA
