# Supplementary material for: Robust classification of protein variation using structural modelling and large-scale data integration
Source: Nucleic Acids Res. 2016 Feb 28;44(6):2501–13. doi: 10.1093/nar/gkw120 (PMC4824117; doi:10.1093/nar/gkw120)
Supplement: SUPPLEMENTARY DATA [file supp_gkw120_nar-00426-z-2016-File002.pdf]

Supplementary Material

1. **Datasets**
2. **Supplemental Files**
3. **Performance Metrics**
4. **VTS Acquisition and Curation**
   - 4.1 **Label Curation**
   - 4.2 **Acquiring Structural Models and Homology Models**
   - 4.3 **Structure-based Features From Rosetta Analysis**
   - 4.4 **VIPUR Software Implementation and Availability**
5. **Assessment of VIPUR Classifier Training and Performance**
   - 5.1 **Feature Selection Using Sparse Logistic Regression**
   - 5.2 **VIPUR Performance Breakdown and Biases**
   - 5.3 **VIPUR Avoids Confounding Sources of Circular Predictions**
6. **Comparison to Other Prediction Methods**
   - 6.1 **Comparison to Human Variant Classifiers**
   - 6.2 **Obtaining PROVEAN Predictions and Ranking**
   - 6.3 **Training and Optimizing a Support Vector Machine Classifier**
7. **Comparison of Prediction Methods on *De Novo* Mutations Associated with Autism Spectrum Disorders**
8. **Additional Methods Details**
   - 8.1 ***Aminochange* Groups**
   - 8.2 **Definitions of Surface and Buried Positions**
   - 8.3 **Identification of "Essential" Positions**

Supplementary Figures and Tables

- **Table S1. Datasets**
- **Table S2. Performance Trends**
- **Table S3. VIPUR Avoids Circularity**
- **Table S4. Prediction Performance on Human Variants in VTS**
- **Table S5. VIPUR Final Model Selected Features**
- **Figure S1. VTS Curation**
- **Figure S2. VIPUR Features**
- **Figure S3. Pairwise Correlation Between Features**
- **Figure S4. Feature Selection and Generalization**
- **Figure S5. Structure-only Feature Selection and Generalization**
- **Figure S6. ROC and PR Curves**
- **Figure S7. ROC and PR Curves on Human Variants**
- **Figure S8. ROC and PR Curves on Non-Human Variants**
- **Figure S9. Parameter Optimization for a Radial-Basis Function Support Vector Machine using LibSVM**
- **Figure S10. V117M Disrupts Strand Pairing for ADIPOQ**
- **Figure S11. W11R Eliminates a DNA Contact**
- **Table S6. ClinVar Predictions**
- **Figure S12. ClinVar Predictions**
- **Figure S13. VIPUR Deleterious Predictions Identify Autism-associated Mutations**
- **Figure S14. Simons Simplex Collection Proband Enrichment is Robust to the Bin Size**
- **Figure S15. Simons Simplex Collection Proband Enrichment is Robust to the Score Threshold**

1 Datasets

dataset	variants	positive label	# of variants	negative label	# of variants	label bias
**VTS**	9,477	deleterious	5,740	neutral	3,737	1.53
Human Outgroup	1,542	deleterious	1,051	neutral	491	2.14
Natural Variant Human Outgroup	383	deleterious	311	neutral	72	4.32
shared Human Outgroup	950	deleterious	664	neutral	286	2.32
Non-Human Variants	4,992	deleterious	3,471	neutral	1,521	2.28
ClinVar	2,295	pathogenic	1,797	benign	498	3.61
Simons Simplex Collection	2,226	found in proband	1,335	found in sibling	891	1.50

Supplementary Table S1: **Datasets**
A summary of all datasets used, their positive and negative labels, counts, and label bias. Every variant in these datasets has a structural model (PDB).

- **VTS** - the VIPUR training set, curated from HumDiv and UniProt (using the curation rules of HumDiv), a large number of these mutations are derived from mutagenesis experiments which may more closely resemble *de novo* mutations (as opposed to variants found in an existing population)
- **Human Outgroup** - a subset of **VTS** that includes only variants found in human proteins and excludes HumDiv, for the purposes of having an outgroup to compare generalization error with PolyPhen2 (which is trained using HumDiv), in this comparison, another VIPUR-like classifier (VIPUR*) is used
- **Natural Variant Human Outgroup** - a subset of the Human Outgroup set that includes only variants that have been identified in the human population (`VARIANT` field in UniProt records), for the purposes of having an outgroup to compare generalization error on a set of human variants more closely resembling the specific training sets of other variant annotation methods
- **shared Human Outgroup** - a subset of the Human Outgroup set that includes variants which have predictions available in dbNSFP for the variant annotation methods SIFT and CADD, for the purposes of having an outgroup to compare generalization error with additional methods (the full Human Outgroup dataset is notably larger and used to compare methods with predictions available)
- **Non-Human Variants** - a subset of **VTS** that includes variants in non-human proteins, for the purposes of comparing prediction performance on variants in other species, including PROVEAN and PolyPhen2 predictions, it should be noted that PolyPhen2 is not designed to predict variants in non-human proteins
- **ClinVar** - the subset of ClinVar variants with reliable structural models, used to compare VIPUR deleterious and neutral labels to the biological annotations of pathogenic and benign, additional predictions on a set of variants in ClinVar with uncertain significance are described in Figure 3
- **Simons Simplex Collection** - the subset of the Simons Simplex Collection with reliable structural models, consists of *de novo* variants found in children with Autism-Spectrum Disorders and their siblings with no intellectual disability phenotype, for the purposes of comparing the predictions made by VIPUR and other variant annotation methods on variants without clear causal effects, deleterious/damaging predictions are expected to be enriched for proband mutations (e.g. probands have a higher deleterious load)

2 Supplemental Files

2.1 VTS

- `VIPUR_training_set.with_meta_data.tsv`

  a tabular text file summarizing the variants in **VTS**, including training features, GO annotations, label curation details, homology modeling information, and several other features

- `VIPUR_training_set.features.tsv`

a tabular text file summarizing the features generated for variants in **VTS**, conveniently available for data mining the VIPUR features and learning new classifier models

- `VIPUR_training_set.predictions.tsv`

  a tabular text file summarizing the predictions made by VIPUR for variants in **VTS**, including the structure-only and sequence-only predictions, top ranking features for deleterious predictions, and weighted terms used in making the prediction

- `VIPUR_training_set_structures.tar.gz`

  a compressed directory (tar gzip) of the input structures used for **VTS**

- `Human_Outgroup_from_VTS.tsv`

  a tabular text file summarizing the variants in the Human Outgroup dataset, including the curated label and predictions made by VIPUR*, PolyPhen2 (HumDiv), and PROVEAN

- `Human_Natural_Variant_Outgroup_from_VTS.tsv`

  a tabular text file summarizing the variants in the Human Natural Variant Outgroup dataset, including the curated label and predictions made by VIPUR*, PolyPhen2 (HumDiv), and PROVEAN

- `Human_Outgroup_with_DBNSFP_predictions_from_VTS.tsv`

  a tabular text file summarizing the variants in the shared Human Outgroup dataset, including the curated label and predictions made by VIPUR*, PolyPhen2 (HumDiv), PROVEAN, SIFT, and CADD

- `Non_Human_Variants_from_VTS.tsv`

  a tabular text file summarizing the variants in Non-Human proteins in **VTS**, including the curated label and predictions made by VIPUR, PolyPhen2 (HumDiv), and PROVEAN

2.2 VIPUR Weights

- `final_classifier.weights`

  a text file listing the weights learned by VIPUR, the first number is the intercept/bias and successive lines correspond to each of the 106 VIPUR features (e.g. the second line is the weight for the first feature etc.)

- `sequence_only.weights`

  a text file listing the weights learned by the sequence-only classifier, the first number is the intercept/bias and successive lines correspond to each of the 106 VIPUR features (e.g. the second line is the weight for the first feature etc.), the first five features are the "sequence" features

- `structure_only.weights`

  a text file listing the weights learned by the structure-only classifier, the first number is the intercept/bias and successive lines correspond to each of the 106 VIPUR features (e.g. the second line is the weight for the first feature etc.), all features after the first five features are the "structure" features

- `training_set_features.means`

  a text file listing the means for each of the 106 VIPUR features in the training set, used to normalize raw input features

- `training_set_features.stds`

  a text file listing the standard deviations for each of the 106 VIPUR features in the training set, used to normalize raw input features

- `VIPUR_HumDiv_trained_only.weights`

  a text file listing the weights learned by the VIPUR* classifier, using the 20 features selected for VIPUR with weights learned by training on variants in HumDiv and non-human proteins (for providing a true generalization comparison on the Human Outgroup datasets)

2.3 ClinVar

- `clinvar_input_structures.tar.gz`

  a compressed directory (tar gzip) of the input structures for predicting the effects of variants in the ClinVar dataset

- `clinvar_output_best_structures.tar.gz`

  a compressed directory (tar gzip) of the best (lowest scoring) output structures for predicting the effects of variants in the ClinVar dataset

- `ClinVar_VIPUR_raw_features.tsv`

  a tabular text file summarizing the features generated for variants in the ClinVar dataset, conveniently available for data mining the VIPUR features and learning new classifier models

- `ClinVar_VIPUR_full_predictions.tsv`

  a tabular text file summarizing the predictions made by VIPUR for variants in the ClinVar dataset, including the structure-only and sequence-only predictions and top ranking features for deleterious predictions

- `ClinVar_DBNSFP_and_other_prediction_scores.tsv`

  a tabular text file summarizing the variants in the ClinVar dataset, including predictions made by VIPUR, PolyPhen2 (HumDiv), PROVEAN, SIFT, and CADD

2.4 Simons Simplex Collection

- `ssc_input_structures.tar.gz`

  a compressed directory (tar gzip) of the input structures for predicting the effects of variants in the Simons Simplex Collection

- `ssc_output_best_structures.tar.gz`

  a compressed directory (tar gzip) of the best (lowest scoring) output structures for predicting the effects of variants in the Simons Simplex Collection

- `Simons_Simplex_Collection_full_VIPUR_predictions.tsv`

  a tabular text file summarizing the predictions made by VIPUR for variants in the Simons Simplex Collection, including the structure-only and sequence-only predictions and top ranking features for deleterious predictions

2.5 VIPUR Executables

Compiled executables for running the VIPUR pipeline can be found in `VIPUR_feature_executables.tar.gz`.

- `psiblast_blast+2.2.25`
- `relax_r54167.64bit.linuxgccrelease`
- `ddg_monomer_r54167.64bit.linuxgccrelease`
- `score_r54167.64bit.linuxgccrelease`
- `rosetta_database`
- `probe`

Rosetta executables are architecture specific and are provided for a 64bit Linux system using gcc.

3 Performance Metrics

Several metrics are used to evaluate the performance of prediction methods on samples with known labels. A dataset with binary labels consists of samples with either the Positive Condition or the Negative Condition. Here, the Positive Condition corresponds to a "**deleterious**" or "**pathogenic**" label and the negative condition corresponds to a "**neutral**" or "**benign**" label.

- *Positive Condition* - the property being predicted, here, the **deleterious** or **pathogenic** label
- *Negative Condition* - the mutually exclusive category for the Positive Condition, here, the **neutral** or **benign** label
- *Label Bias* - the ratio of the number of samples that have the Positive Condition to the number of samples that have the Negative Condition, describes the default expectation for samples drawn from a dataset (Positive if Label Bias > 1, Negative if Label Bias < 1)

A binary classifier will produce either a Positive Prediction or a Negative Prediction corresponding to predictions of the Positive or Negative Conditions. A set of predictions can be described by the number of correct (Condition and Prediction match) and incorrect (Condition and Prediction do not match) predictions made for both Positive and Negative Predictions yielding:

- *True Positives* - samples that have the Positive Condition and a Positive Prediction (correct), described by the number of these samples, TP
- *False Positives* - samples that have the Negative Condition and a Positive Prediction (incorrect), described by the number of these samples, FP
- *True Negatives* - samples that have the Negative Condition and a Negative Prediction (correct), described by the number of these samples, TN
- *False Negatives* - samples that have the Positive Condition and a Negative Prediction (incorrect), described by the number of these samples, FN

We provide these counts or their scaled versions in several tables (scaled by the number of samples in the associated Condition ex. TPR is the *True Positive Rate*, $TPR = TP/(TP + FN)$).

To compare between classifiers and datasets of different sizes, these fundamental counts are used to calculate additional performance metrics (often fractions of the different Condition or Prediction labels).

- *Accuracy* - the fraction of correct cases, $ACC = (TP + TN)/(TP + FP + TN + FN)$
- *Balanced Accuracy* - a performance metric considering the average of correct prediction rates for both Positive and Negative categories, equivalent to "balancing" the label bias, $BAL\_ACC = .5 * TP/(TP + FN) + .5 * TN/(TN + FP)$

4 VTS Acquisition and Curation

4.1 Label Curation

We collected protein variants (nonsynonymous SNPs) from HumDiv[1] and UniProt[2] with clear deleterious or neutral effects. These variants were mapped onto crystallographic and comparative models of the protein macromolecules from the Protein Data Bank[4], ModBase[39], and SwissModel[44]. HumDiv is a database of naturally occurring human protein variants annotated as causing Mendelian diseases and used for calibration and testing numerous prediction tools[1]. We extracted additional variant annotations from UniProt[2] using the curation rules of HumDiv. Variants with annotations describing clear evidence that some molecular activity essential to the protein function is disrupted are labeled '**deleterious**' (if the activity is reported, only activity $\leq 5\%$ is labeled deleterious). Variants with annotations describing clear evidence that all known molecular activities essential to the protein function are unperturbed are labeled '**neutral**' (if the activity is reported, only activity $\geq 70\%$ is labeled neutral). If there is insufficient evidence, we do not assign either label, including annotations with clear effects that do not guarantee disruption of molecular function (ex. no annotation, disease-associated, lethality, low expression, improper localization, etc.).

4.2 Acquiring Structural Models and Homology Models

We searched for crystal structures and comparative models of proteins in the dataset to maximize coverage. For proteins present in HumDiv without crystal structures in the PDB, we produced comparative models using Modeller[14, 13]. We restricted our templates to structures generated using X-ray crystallography with more than 20% sequence identity to the query protein, selecting templates with the highest sequence identity match to the query. When a single template could not cover all variant positions (e.g. missing densities), multiple models were constructed (e.g. separate domains) or multiple templates were used to cover these missing regions. Comparative models were produced using Modeller for threading[14], skipping refinement steps that would be redundant with refinement during feature generation. Many of these comparative models had obvious structural defects, such as broken loops and improbable backbone Φ-Ψ angles, requiring curation of over 5,000 putative models. For proteins with sufficient variant annotation in UniProt but without structures in the PDB, we extracted comparative models from ModBase[39] and SwissModel[44] (no restriction on the template sequence identity to query), selecting models with the largest sequence identity match to the query. All protein models were standardized to remove unwanted coordinates (duplicate chains, ligands, metals, and non-standard amino acids). We removed all structures covering transmembrane regions since our current Rosetta analysis does not appropriately sample or score transmembrane regions. This curation process resulted in 9,477 variants in 2,637 models of 2,444 proteins (see Supplementary Figure S1).

4.3 Structure-based Features From Rosetta Analysis

The `ddg_monomer` protocol is designed to approximate the change in free energy upon mutation ($\Delta\Delta G$) and uses a fast refinement protocol which outputs the change in Rosetta Energy (stability)[20], contributing 17 features to our analysis. Rosetta FastRelax uses Monte Carlo sampling of protein backbone conformations with side chain optimization[49] to find low Energy conformations. We run Rosetta FastRelax to generate 50 low Energy conformations[20] for both the native and variant proteins. We include additional features describing the geometric differences between the input and final structure for each trajectory (e.g., RMSD and gdtmm) to detect proteins undergoing large rearrangements, totaling 23 features. To compare the native and variant ensembles and eliminate potential differences in score magnitude across diverse protein folds, we 1) extract the distributions of each Rosetta score term for the native and variant proteins, 2) calculate the quartiles of the variant protein score distributions, and 3) calculate the cumulative density for these quantiles on the corresponding native protein score distribution[40]. FastRelax and quartile analysis produce three features per score term for each variant, corresponding to the Q1, Q2, and Q3 quartiles[40], totaling 60 features.

4.4 VIPUR Software Implementation and Availability

VIPUR is currently available as an independent Python module and requires BLAST+, ROSETTA, and PROBE. VIPUR runs on a structural model of the native protein structure (in PDB format) and a file containing the variants to predict (e.g. S204P), or an entire directory of these files. VIPUR verifies the positions and native amino acids of the variant file and controls execution of PSIBLAST[5], Rosetta `ddg_monomer`[20], Rosetta FastRelax[49], and PROBE[52]. The output includes predictions from the VIPUR classifier and classifiers using only structure-based features and sequence-based features, and an interpretation of the variant effect including the top ranking structural features. Please see the VIPUR code for full usage and analysis details, available at `https://osf.io/bd2h4`.

5 Assessment of VIPUR Classifier Training and Performance

5.1 Feature Selection Using Sparse Logistic Regression

VIPUR uses logistic regression as a statistical classification framework to robustly discriminate between **deleterious** and **neutral** protein variants from the derived 106 sequence- and structure-based features. Logistic regression generalizes linear regression to binary classification by linking known class labels (y_i, **deleterious** vs. **neutral**) to our feature set (vectors $\mathbf{x}_i$) using a logistic function and allows for a natural probabilistic interpretation of the classification outcome ($\text{Prob}(y_i|\mathbf{x}_i)$). Consider our dataset by $X \in \mathbb{R}^{p\ x\ n}$ where $p = 106$ is the number of features and $n = 9,477$ denotes the number of labeled variants. For each column (variant) characterized by features $\mathbf{x}_i \in X$ we have a binary class label $y_i \in [-1, 1]$ where the positive label indicates a **deleterious** variation. These class labels, derived from curated annotations, are stored in the vector $y \in \mathbb{R}^n$. We learn

$$\text{Prob}(y_i|\mathbf{x}_i) = \frac{1}{1+\exp(\mathbf{w}^T\mathbf{x}_i + c)} \tag{1}$$

where $\text{Prob}(y_i|\mathbf{x}_i)$ estimates the conditional probability of label y_i given the sample $\mathbf{x}_i$. The model is characterized by a weight vector $\mathbf{w} \in \mathbb{R}^p$ and bias/intercept $c \in \mathbb{R}$. This model provides separation by learning the $\mathbf{w}^T\mathbf{x}_i + c = 0$ hyperplane in the feature space, where $\text{Prob}(y_i|\mathbf{x}_i) = 0.5$. Thus, $\mathbf{w}^T\mathbf{x}_i + c > 0$ corresponds to a **deleterious** prediction with individual weight w_j corresponding to the relative importance of the feature for making this separation. We determine $\mathbf{w}$ by finding the minimum of the associated negative log-likelihood (also called the logistic loss) on the training data $\{\mathbf{x}_i, y_i\}_{i=1}^m$.

$$f(\mathbf{w}, c) = \frac{1}{m}\sum_{i=1}^{m} \log(1 + \exp(-y_i(\mathbf{w}^T\mathbf{x}_i + c))) \tag{2}$$

To arrive at a classifier with a minimal, non-redundant feature set that simultaneously generalizes well across all protein variants, we repeatedly split the dataset into training and test sets (100 random splits) and compute logistic regression models across all model complexities (i.e., using one to 106 features). We use sparsity-promoting L1-regularization[33, 26] to reduce complexity, seeking the minimum of this loss function, $f(\mathbf{w}, c)$ while simultaneously promoting sparsity of the weight vector $\mathbf{w}$ as described below:

$$f_S(\mathbf{w}, c) = f(\mathbf{w}, c) + \lambda\,|\mathbf{w}|_1\,, \tag{3}$$

where $|\cdot|_1$ denotes the L1 norm and $\lambda > 0$ is a parameter promoting sparsity (tunable). This sparsity implies that only a few features are used to predict the class labels (most weights w_j "shrink" to 0). For any fixed λ the convex non-smooth loss $f_S(\mathbf{w}, c)$ can be efficiently solved using projected sub-gradient methods.

The sparsity constraint restricts the number of features selected during training, but does not ensure features are robustly selected. Similar to stability selection in linear regression [31], we recorded the frequency of features that are present in the best predictive model on each test set. We determined the model complexity (number of features) that maximizes the average generalization performance to be 20 and thus select the 20 most frequent features (Figure 2B, Supplementary section 5.1). Samples were split by proteins, rather than variants, to prevent the classifier from learning protein-specific patterns[1]. For each split, we tuned the sparsity constraint (parameter λ) and selected the model that minimized error on the testing set. We generated and tested 100 of these splits and recorded the features selected for every model (Supplementary Figure S4). Features were ranked according to their prevalence in the trained models of these splits, selecting 20 features that maximize generalization performance (Figure 2, Supplementary Figure S4) and perform better than models trained on all 106 features. We tested generalization performance for classifiers trained on increasing fractions of the full dataset to determine robustness. Classification and generalization performance approach very similar values when trained on only 50% of the data, demonstrating models learned from these 20 are generalizable and not overfit (Figure 2B, Supplementary Figure S4). The final VIPUR classifier was trained on the full dataset using only these 20 selected features. We evaluated the performance of the final sparse logistic regression classifier on 100 independent random splits (80% training, 20% testing) by means of average Precision-Recall (PR) and Receiver-Operating Characteristic Curves (ROC) (Figure 2, Figure S6). VIPUR performs better than several alternative methods, including an optimized SVM with a radial basis function kernel (Supplementary Figure S9).

5.2 VIPUR Performance Breakdown and Biases

To ensure our classifier was trained on a sufficiently diverse set of protein variants, we integrated naturally occurring variation with variants produced by mutagenesis and pseudomutations derived from differences between humans and closely related mammals[1]. There is an abundance of protein-specific data available for the proteins in our dataset, however, we restricted our features to information/analyses available for under-researched proteins (hence benchmarking with comparative model structures). We investigated prediction trends of VIPUR for numerous protein properties including the source of data, species of origin, structural context, functional annotation, and model quality to identify any biases in our predictions and suggest which sources of information may improve VIPUR further. For each of these protein "subsets", we tested if performance on these variants had biased accuracy, error rates, or composition. We considered groups based on: data source (HumDiv or UniProt), model source, domains, species, structural context (surface, core etc.), GO molecular function, GO biological process, and amino acid transitions using a Pearson chi-squared test, restricting our inquiry to groups with at least 100 samples in the data. Here, we comment on the most significant deviations, correcting for cases where skewed predictions occurred on imbalanced

samples (ex. surface variants have a higher number of neutral predictions and also have a higher number of **neutral** samples).

				Structure		Sequence	
Subset	Variants	FPR	FNR	FPR	FNR	FPR	FNR
All Samples	9477	0.17	0.23	0.26	0.36	0.20	0.26
†Low Quality Comparative Models	921	+0.06	**+0.10**	+0.07	**+0.13**	+0.08	+0.05
Yeast (*S. cerevisiae*)	372	**+0.10**	+0.03	+0.07	**+0.14**	**+0.10**	+0.03
A. thaliana	292	+0.04	**+0.10**	+0.02	**+0.19**	+0.04	+0.06
Endonuclease Activity (GO:0004519)	209	**+0.10**	+0.09	+0.04	**+0.15**	+0.09	+0.04
mRNA processing (GO:0006397)	150	**+0.19**	-0.09	**+0.19**	-0.05	**+0.17**	**-0.18**
Sequence-Specific DNA Binding (GO:0043565)	103	**+0.17**	-0.02	**+0.17**	-0.06	**+0.25**	-0.01
DNA-Dependent Transcription (GO:0006355)	598	+0.05	**+0.10**	+0.05	**+0.18**	+0.05	+0.07
DNA Binding (GO:0003677)	604	**+0.10**	+0.05	**+0.10**	**+0.12**	**+0.10**	0.00
Ligase Activity (GO:0016874)	213	+0.01	0.00	+0.02	**+0.28**	-0.01	-0.06
Lyase Activity (GO:0016829)	380	-0.03	**+0.14**	-0.08	**+0.34**	-0.06	**+0.10**
Iron-Sulfur Cluster Binding (GO:0051536)	144	-0.03	**+0.27**	-0.05	**+0.46**	-0.06	**+0.19**
4 Iron, 4 Sulfur Cluster Binding (GO:0051539)	123	-0.07	**+0.32**	**-0.12**	**+0.47**	-0.11	**+0.21**
Transition to R	640	-0.07	0.00	-0.06	-0.03	-0.09	0.00
Transition to Y	172	**-0.10**	+0.01	-0.08	-0.02	**-0.14**	+0.02
Transition to H	260	-0.05	**+0.14**	+0.01	+0.03	-0.02	**+0.20**
Transition to G	285	+0.09	-0.07	+0.03	**-0.16**	**+0.10**	-0.03
Transition from E	685	+0.02	+0.09	-0.01	**+0.10**	0.00	+0.06
Transition to E	366	-0.03	+0.07	+0.01	**+0.11**	-0.03	+0.01
Transition T to A	173	+0.05	-0.05	**+0.17**	+0.09	+0.06	-0.08
Transition E to Q	160	-0.04	**+0.14**	**-0.10**	**+0.27**	-0.07	**+0.10**
Transition D to N	240	-0.05	**+0.13**	-0.09	**+0.26**	-0.04	**+0.17**
Transition Y to F	118	-0.01	+0.07	**+0.22**	**+0.24**	-0.01	+0.03

Supplementary Table S2: **Performance Trends**
FPR = False Positive Rate, FNR = False Negative Rate, †comparative models from templates with < 30% sequence identity to the query
Error Rates more than .10 above expectation are in **bold**

There is no identifiable difference in performance between variants from HumDiv and UniProt despite HumDiv containing solely human data. Surprisingly, we also detect no difference in performance between models drawn from the PDB, ModBase, SwissModel, or produced by Modeller (not shown). As expected, lower quality comparative models perform worse than high quality models (models from templates under 30% sequence identity are reduced to 73% accuracy). These lower quality models have higher false negative rate, suggesting that low quality models do not capture the interactions near the variant position that are destabilized upon mutation. We do not observe a difference in performance between crystal structures and high quality comparative models, although model defects may already be masked by our structure-based features which always consider the differences between native and variant models. Our dataset contains examples for 323 of the 400 possible canonical amino acid transitions and we detect a slightly higher false negative rate for transitions to histidine and transitions from glutamic acid to lysine.

Across organismal domains, eukaryotic proteins have a slightly increased false negative error rate while prokaryotic proteins have a slightly increased false positive rate. This trend may be caused by label imbalance as the majority of **neutral**-labeled variations are eukaryotic. Human proteins are predicted more accurately that other species, likely due to label imbalance since most **neutral** annotations are in human proteins. Proteins from yeast (*S. cerevisiae*) and *A. thaliana* have slightly higher error rates than expected. We do not detect any difference in performance for variants on the surface or interior of proteins, although labels and predictions are both imbalanced with more **neutral** examples on the protein surface and more **deleterious** examples in the protein core.

We observe a higher than expected false positive rate among proteins with DNA binding functions including sequence-specific DNA binding (GO:0043565), DNA-dependent transcription (GO:0006355), and cell cycle (GO:0007049) annotations. Although we expected the structure-based features to perform worse for proteins associated with nucleic acids (due to the absence of these molecules in the structural model), this functional bias comes from both feature sets. A similar bias occurs for transitions to glycine and may represent a tendency to classify all variations at highly conserved sites as **deleterious**, independent of the new amino acid's properties. We do not observe biases for other nucleic acid functions (nucleotide binding, RNA binding, nuclease activity) however, we see a higher false positive

rate for endonuclease activity (GO:0004519) and mRNA processing (GO:0006397) annotations in both feature sets. The structure-based features have higher false negative rates for catalytic activity (GO:0003824), specifically ligase (GO:0016874) and lyase (GO:0016829) activity annotations, however, all of these trends are reduced in the combined classifier.

We anticipated that many active sites in our structural models would lack chemical interactions necessary for correct classification since ligands and cofactors are not included. However, several of these positions have highly constrained conformations that allow accurate **deleterious** predictions with structure-based features even without the ligand present (Figure 5). We also observe an increased false negative rate for iron-sulfur associated proteins (GO:0051536, GO:0051539), and a slight decrease in performance for metal binding proteins (GO:0046872). As with ligands, this decrease in performance was much less than anticipated (Supplementary Table S2), likely caused by the highly constrained geometries of metal coordination sites that are captured by our structure-based features, even without the metal(s) present. We observe several amino-acid specific biases, mostly for the structure-only classifier, indicating areas for improvement. Our classifier predicts mutation to arginine and to tyrosine correctly for nearly all examples available. The structure-based features have a higher false positive rate for the transitions: glutamic acid to glutamine, aspartic acid to asparagine, threonine to alanine, and tyrosine to phenylalanine. These transitions may involve subtle water coordination sites, and generally indicate that performance could be improved with more rigorous electrostatics methods.

Nearly all of the trends for sequence-based features involve higher false positive rates while trends for the structure-based features involve higher false negative rates. Since mutations at conserved positions are more likely to be **deleterious** than non-conserved positions, sequence-based analysis is more likely to falsely label variation at conserved sites as **deleterious** (too sensitive), independent of the new amino acid's properties. Since our structural models do not include binding partners (proteins, DNA, ligands, metals), this structure-based analysis is missing some interactions and limited to predicting **deleterious** variants that cause energetic disruption of the monomer. When combined, these two sources of information provide a clear interpretation of conservation and destabilization, allowing the combined confidence metric to scale directly with performance and correctly classify variants missed by both feature sets independently. Many of these trends suggest areas for improvement, particularly the use inclusion of nucleic acid models and more sophisticated electrostatic methods for polar amino acid characterization.

5.3 VIPUR Avoids Confounding Sources of Circular Predictions

A recent study of pathogenicity prediction methods showed that several classifiers trained on mutational data made circular predictions, effectively learning a majority vote rule for specific protein families[16]. This circularity highlighted the difficulty to learn generalized classifiers on data with a high label bias (e.g. when there are many more **deleterious** samples than **neutral** ones) and suggested methods for avoiding and testing for circularity. Unlike other similar classifiers, VIPUR was trained on a highly curated set of variants with experimentally validated labels. This curation process eliminated many variants with ambiguous effects, limiting the number of proteins that contribute multiple mutations and reducing the label bias to $\approx 3/5$ (5,740 deleterious variants and 3,737 neutral variants).

To prevent any circularity from inflating evaluation of VIPUR, we ensured that all divisions of training and testing sets were striated by the protein identity, such that all variants from any individual protein are contained only in a single training or testing set (e.g. never training and testing on variants from the same protein). This guarantees that our performance metrics are evaluated on variants in proteins that had never been seen before. Assessment of circularity can be done by investigating any performance differences between variants in proteins with only a single variant in the training set and those with multiple[16]. **VTS** contains 1,125 'single' variants in proteins that contain no other variants in the dataset, 3,475 'pure' variants in proteins with multiple variants that all share the same label, and 4,877 'impure' variants in proteins with multiple variants containing at least one from both labels (**deleterious** and **neutral**). Both the 'single' and 'pure' categories are enriched for **deleterious** labels while the 'impure' variants are enriched for **neutral** labels, suggesting an interesting bias in the data; that **neutral** samples are rarely provided without accompanying **deleterious** samples. Confounding circularity occurs when training on pure variants leads to overestimation on single and impure variants, creating a classifier that does not properly generalize. VIPUR performance on each of these variant groups is nearly identical to the others and the overall assessment (Supplementary Table S3). As the proportion of **deleterious** and **neutral** variants narrows for 'impure' proteins, there is a notable drop in performance metrics, particularly AUPR, however this likely stems from the high label bias in these subsets (mostly neutral samples). We also compared these performance trends to a simple per protein Majority Vote classifier where variants were simply assigned the majority label of other variants in the same protein ('pure' and 'single' variant predictions are uninteresting). This classifier has a similar trend of decreasing performance as the stricter label ratios are imposed, however even the best majority vote prediction has a similar performance

to the worst performing subset of VIPUR predictions. These methods of assessing circularity are useful but may over-penalize any supervised learning classifier. VIPUR predictions have similar performance trends for variants with many identical labels in the dataset and variants in proteins that have never been seen before, avoiding overfitting by circularity.

	total	del	neu	TPR	TNR	FPR	FNR	acc	AUC	AUPR
all	9477	5740	3737	.86	.73	.27	.14	.81	.86	.88
single	1125	925	200	.88	.49	.51	.12	.81	.77	.92
degenerate	8352	4815	3537	.86	.74	.26	.14	.81	.83	.87
pure	3475	2705	770	.86	.61	.39	.14	.80	.79	.92
impure .5	4877	2110	2767	.86	.78	.22	.14	.81	.84	.51
impure .4	1711	441	1270	.81	.76	.24	.19	.77	.76	.63
impure .3	1192	349	843	.80	.72	.28	.20	.75	.76	.62
impure .2	791	242	549	.84	.68	.32	.16	.73	.60	.58
				Majority Vote						
impure .5	4877	2110	2767	.88	.64	.36	.12	.74	.94	.50
impure .4	1711	441	1270	.50	.72	.28	.50	.66	.64	.37
impure .3	1192	349	843	.53	.63	.27	.47	.60	.67	.35
impure .2	791	242	549	.55	.56	.44	.45	.56	.83	.32

Supplementary Table S3: VIPUR Avoids Circularity
single: variants in proteins that contain no other variants, degenerate: variants in proteins with at least one other variant, pure: variants in proteins with at least one other variant with all variants sharing the same label, impute X: variants in proteins with at least one other variant and a label ratio of .5 ± X

6 Comparison to Other Prediction Methods

6.1 Human Variant Classifiers

PolyPhen2 and many popular pathogenicity detection methods are restricted to classification of human protein variants. We designed VIPUR to run on variants found in any species and avoid overfitting any particular organism. Eliminating this overfitting is particularly important when classifying *de novo* mutations since these variants are likely to be absent from multiple sequence alignments, reducing the accuracy of sequence-based analysis.

We cannot properly compare performance between VIPUR and PolyPhen2 on the full **VTS** since it contains variants in non-human proteins and variants from PolyPhen2's training set (HumDiv). There are 1,542 human variants in **VTS** that are not included in HumDiv and to ensure a fair comparison with PolyPhen2, we retrained a VIPUR classifier (VIPUR*) on the remaining 7,935 variants of **VTS**. We calculated ROC and PR for VIPUR*, PolyPhen2, and PROVEAN on this set of 1,542 variants, a subset of 383 variants found naturally in the human population (383 variants), and 950 variants that also have predictions available for the additional methods SIFT and CADD. We also compare performance of VIPUR, PolyPhen2, and PROVEAN on a set of 4,992 variants found in non-human species.

VIPUR* produces ROC curves similar to PROVEAN and PolyPhen2 with notably higher specificity and a slightly reduced sensitivity (ROC and PR curves cross, Supplementary Figure S7). PROVEAN and PolyPhen2 perform very similarly although PolyPhen2 predictions are restricted to a small region of the PR landscape since many predictions obtain scores of '0' or '1' (Supplementary Figure S7A,B). While this limitation of PolyPhen2 scores restricts the inferences on PR performance, it reflects a practical limitation caused by degeneracy of the output metric. Across 1,542 human variants, VIPUR* has higher AUROC (Supplementary Figure S7A) compared to PROVEAN and PolyPhen2 with a notably higher AUPR (Supplementary Figure S7B). These variants have a notable label bias and are drawn primarily from mutagenesis of human proteins, resembling *de novo* mutations.

When performance is evaluated on a subset of 383 variants (with notably higher label bias) that are found within the human population, PROVEAN and PolyPhen2 performance increases notably, although VIPUR*'s ranking ability remains superior (Supplementary Figure S7C,D). These 1,542 variants are included in the training set of VIPUR to ensure it generalizes to mutagens and to eliminate artificially inflated performance metrics.

1,542 variants (1,051 deleterious, 491 neutral, 2.14 label bias)								
	TP	FN	FP	TN	Acc	Bal Acc	AUROC	AUPR
VIPUR*	888	166	199	289	0.762	**0.716**	**0.777**	**0.846**
PROVEAN	973	78	265	226	**0.778**	0.693	0.745	0.796
PolyPhen2	992	59	306	185	0.763	0.66	0.749	0.727
383 variants (311 deleterious, 72 neutral, 4.32 label bias)								
VIPUR*	249	65	22	47	0.767	0.730	0.797	**0.929**
PROVEAN	279	32	27	45	0.846	**0.761**	**0.810**	0.914
PolyPhen2	291	20	34	38	**0.859**	0.732	0.805	0.858
950 variants (664 deleterious, 286 neutral, 2.32 label bias)								
VIPUR*	548	112	91	191	0.782	**0.752**	**0.801**	**0.877**
PROVEAN	615	49	148	138	**0.793**	0.704	0.757	0.823
PolyPhen2	626	38	163	123	0.788	0.686	0.77	0.748
SIFT	588	76	135	151	0.778	0.707	0.76	0.76
CADD	605	59	149	137	0.781	0.695	0.757	0.842

Supplementary Table S4: **Prediction Performance on Human Variants in the VTS**
TP = True Positives, FN = False Negatives, FP = False Positives, TN = True Negatives, Acc = Accuracy, Bal Acc = Balanced Accuracy (equal weight for both label classes), AUROC = Area Under the Receiver-Operating Curve, AUPR = Area Under the Precision-Recall curve

VIPUR outperforms the additional methods SIFT and CADD over a subset of human variants with predictions available for all methods (950 variants, Supplementary Figure S7E,F). The accuracy, balanced accuracy, AUROC, and AUPR for PolyPhen2 and PROVEAN are very similar to the full 1,542 human variant evaluation, though slightly improved. VIPUR similarly performs better than the full human subset, suggesting the slightly increased label bias may account for these differences. SIFT and PolyPhen2 achieve very similar performance statistics. CADD demonstrates a high ranking ability (AUPR .84) despite having very similar accuracy and AUROC measures to PROVEAN, PolyPhen2, and SIFT.

Although PolyPhen2 and PROVEAN are not trained to predict the effects of variants in non-human species, they both perform well on a set of non-human variants. To ensure this comparison does not over-inflate any organism-specific contribution to performance, we calculated averaged ROC and PR curves on 100 random samples of 1000 variants, split by protein (from a set of 4,992 non-human variants). VIPUR outperforms both PolyPhen2 and PROVEAN in ROC performance and has a notably increased AUPR compared to these methods. The relative difference in performance is very similar to the performance difference between VIPUR and PROVEAN on the entire **VTS**, suggesting the diversity of VIPUR's training set contributes to its improved performance. PolyPhen2 achieves a higher AUROC that PROVEAN and even crosses the VIPUR ROC curve, indicating a slightly increased sensitivity in lower scoring predictions. As in the other comparisons, PolyPhen2's output scores have very similar values, hindering comparison of PR performance and demonstrating an inability to distinguish true positives from false positives in high confidence predictions.

6.2 Obtaining PROVEAN Predictions and Ranking

We compared performance of our predictive model to PROVEAN, a highly accurate method for labeling protein variants as "**damaging**" or "**neutral**". For human variants, PROVEAN was run remotely using the (PROVEAN server at `http://provean.jcvi.org/index.php`). For non-human variants, PROVEAN was run locally using CD-hit 4.5.4. The output PROVEAN score does not have a clear increase in accuracy for high scoring predictions. We scaled PROVEAN scores between 0 and 1 over the range of scores obtained on this dataset with 0 being the smallest score and 1 being the largest score. PR and ROC curves are generated from *ranked* predictions so this scaling should have no impact on performance.

6.3 Training and Optimizing a Support Vector Machine Classifier

We compared the performance of our logistic regression classifier to an optimized support vector machine using the entire feature set. Training and testing was performed using LibSVM (version 3.1) through Weka (3.6.0). We used

a radial basis function kernel optimized over C $\in \{2^{-1}, 2^0, 2^1, ..., 2^9\}$ and $\gamma \in \{2^{-8}, 2^{-7}, ..., 2^{-1}\}$. Optimal parameters (C= 2^2, $\gamma = 2^{-4}$) were selected using 5-fold cross-validation.

We evaluated performance using AUPR and AUROC for 100 splits of 80% training and 20% testing. Probability estimates for each prediction are determined in LIBSVM using a form of Platt et al.[17].

7 Comparison of Prediction Methods on De Novo Mutations Associated with Autism Spectrum Disorders

We generated VIPUR predictions for 2,814 *de novo* mutations in the Simons Simples Collection. Predictions for PolyPhen2, CADD, and SIFT were obtained from dbNSFP[28]. To ensure method predictions is comparable, we only used the 2,226 variants that have predictions available for all of these methods. We calculated the enrichment of **deleterious**/intolerant/damaging predictions for proband mutations by comparing the ratio of proband mutations to mutations in unaffected siblings across different score cutoffs. We expect high scoring **deleterious**/intolerant/damaging predictions to be more enriched for proband mutations and low scoring predictions to be enriched for mutations from unaffected siblings. This prediction trend is calculated as the correlation between each method's deleteriousness scores and the enrichment for proband mutations. Proband or sibling association does not establish deleteriousness. However we expect proband mutations to include more **deleterious**/intolerant/damaging predictions, leading to a large positive correlation between the prediction scores of these methods and the proband enrichment. The correlation values reported in the main text are calculated using 10 bins and this parameter will alter the exact correlation values obtained, but not the overall ranking of these methods (Figure S14).

Independent of the number of bins used for calculating the correlation, VIPUR predictions correlate more highly with proband enrichment than other methods (Supplementary Figure S14). Since the number of bins is arbitrary, smaller bin sizes produce increasingly undersampled correlation estimates (fewer samples each bin) with most methods having a notable drop after 10 bins. Several methods do not notably change behaviour between 3 and 10 bins so we have reported Spearman rank correlation and Pearson correlation values using 10 bins. Independent of the threshold used to define deleterious and neutral classifications, VIPUR provides higher enrichment than other methods for **deleterious** predictions and lower or comparable enrichment for **neutral** predictions (Supplementary Figure S15). Most methods fluctuate around the background enrichment ratio, but VIPUR, PolyPhen2, and SIFT all have the expected enrichment trends, with VIPUR notably outperforms the other methods. Deleterious prediction proband enrichment using VIPUR sharply increases above .5 and this threshold is used as the default boundary for **deleterious-neutral** definition to fairly represent VIPUR's enrichment (rather than arbitrarily choosing a scoring scheme where VIPUR outperforms other methods). PolyPhen2 and SIFT follow very similar prediction patterns for these robustness assessments which may suggest a deeper relationship between these methods.

Correlation values were tested for significance using the R software package. The reported p-values are compared to the null hypothesis that each correlation is zero. While BLOSUM62 values provide a very accurate baseline, they are not continuously distributed and can only be separated into 7 different score bins, reducing the overall significance of this correlation. CADD produces several metrics and has no recommended confidence cutoff for identifying deleterious mutations so we used the scaled Raw Rank Score (performs better than CADD Raw Score on this data, available in dbNSFP). We compared predictions on *de novo* mutations to PolyPhen2 HumDiv (rather than PolyPhen2 HumVar) since it is designed to classify rare alleles and shares training data with VIPUR.

8 Additional Methods Details

feature	quartile	method	weight
`bias`			0.710025022037
`aminochange`			0.176334912219
`pssm_mut`		PSIBLAST	-0.433593981328
`pssm_diff`		PSIBLAST	0.588538852189
`info_cont`		PSIBLAST	0.492730459878
`ACCP`		PROBE	-0.121632092225
`total_score`	quartile 1	Rosetta FastRelax	0.270719693437
`pro_close`	quartile 2	Rosetta FastRelax	0.132242051922
`fa_pair`	quartile 1	Rosetta FastRelax	0.0471347304389
`dslf_cs_ang`	quartile 3	Rosetta FastRelax	0.143559326462
`dslf_ss_dih`	quartile 2	Rosetta FastRelax	0.0418810070048
`rama`	quartile 3	Rosetta FastRelax	-0.114677914346
`p_aa_pp`	quartile 3	Rosetta FastRelax	-0.077972713097
`gdtmm3_3`	quartile 1	Rosetta FastRelax	0.134506541874
`gdtmm4_3`	quartile 3	Rosetta FastRelax	-0.171378461317
`ddg_total`		Rosetta *ddg_monomer*	0.0558961760166
`ddg_fa_rep`		Rosetta *ddg_monomer*	0.302094051281
`ddg_fa_sol`		Rosetta *ddg_monomer*	0.118697586386
`ddg_fa_pair`		Rosetta *ddg_monomer*	0.105708851713
`ddg_hbond_bb_sc`		Rosetta *ddg_monomer*	0.0819256780966
`ddg_hbond_sc`		Rosetta *ddg_monomer*	0.0858842832406

Supplementary Table S5: VIPUR Final Model Selected Features - The 20 features used by VIPUR and generated by sequence analysis and structural modeling with Rosetta. The sequence features and Rosetta scores generally representing destabilization (*total_score* and *fa_rep*) are the most informative features and obtain the highest weights. Two *gdtmm* scores obtain curiously high weights with opposite signs, suggesting the overall coordinate differences between the native and variant structures can be informative about either label depending on the parameters used.

8.1 `Aminochange` Groups

We implement a crude "dissimilarity" score termed `aminochange` (from Poultney *et al.*[40]) by comparing the general properties of the native and variant amino acids. Each amino acid is placed into one of seven groups and a substitution is scored "1" if the native and variant amino acids belong to the same group and "2" otherwise. The amino acid groups used for `aminochange` are

- A, I, L, V - Alanine, Isoleucine, Leucine, Valine (small nonpolar)
- C, S, T - Cysteine, Serine, Threonine (small polar)
- D, E - Aspartic Acid, Glutamic Acid (negative charge)
- F, M, W, Y - Phenylalanine, Methionine, Tryptophan, Tyrosine (large nonpolar)
- G, P - Glycine, Proline ("bad behaved")
- H, K, R - Histidine, Lysine, Arginine (positive charge)
- N, Q - Asparagine, Glutamine (side chain amide)

8.2 Definitions of Surface and Buried Positions

The definition of surface and buried positions was taken from a Koga *et al.*(2012)[23], a recent publication using Rosetta for protein design. Residues were classified as "core", "boundary", or "surface" based on their surface area and secondary structure (reduced DSSP representation allowing helix, strand, and loop). Helix and strand residues are considered core if they have a small Solvent Accessible Surface Area (SASA$\leq$ 15Å), surface if they have a large

SASA ($\geq$ 60Å) and boundary if they fall between these thresholds. For loop residues, a larger SASA is tolerated for core ($\leq$ 25Å) and a smaller SASA is required for surface ($\geq$ 40Å). Feature distributions for the boundary residues in our training set appear very similar to the distribution for core residues, while surface residues appear distinct from both. During analysis, we considered both core and boundary classifications as "buried" or "interior" to simplify consideration of local protein environment. The VIPUR code currently assigns these positions based on a 12.5Åcutoff (above this is considered "surface").

	interior		
	core	boundary	surface
helix or strand	SASA$\leq$ 15Å	15Å<SASA< 60Å	SASA$\geq$ 60Å
loop	SASA$\leq$ 25Å	25Å<SASA< 40Å	SASA$\geq$ 40Å

8.3 Identification of "Essential" Positions

The structure-only classifier's confidence metric approximates energetic destabilization, yet many variants in this dataset do not appear **deleterious** due simply to fold destabilization, likely due to the absence of interaction partners in our models. Similarly, the sequence-only classifier confidence metric serves as an appropriate approximation for amino acid conservation. These metrics correlate highly since many, but not all, destabilizing variants occur at conserved positions. Several variant positions where these scores disagree occur at interaction sites, such as W11 at the DNA binding interface of IRF1 (Supplementary Figure S11). At position 11, mutation to arginine eliminates a favorable DNA contact which "abolishes DNA binding" (UniProt annotation). Without DNA in our structural model, this mutation is not detected as destabilizing by the structure-based features even though VIPUR makes a confident **deleterious** prediction (.952), indicating conservation not caused by destabilization of the monomer structure. We observe this same behavior at other binding interfaces, such as the FOXP3 F371C dimer interface (not shown). Even though VIPUR can adequately identify disrupted interactions due to conservation of sequence or structure, incorporating these binding partners into the structural models will improve classification and enhance our automated interpretation of variant effects. Comparing the combined classifier score (destabilization vs conservation) to the structure-only classifier score (just destabilization) can identify "essential" positions that are conserved but *not* due to energetic constraints on the monomer. The precise cutoff for identifying these essential positions is unclear, though they frequently occur when the combined classifier score notably exceeds the structure-only classifier score (at interaction interfaces, score differences frequently exceed .2). The VIPUR code currently identifies potential interaction sites for **deleterious** predictions with a score difference (total - structure-only) of .2.

Supplementary Figures

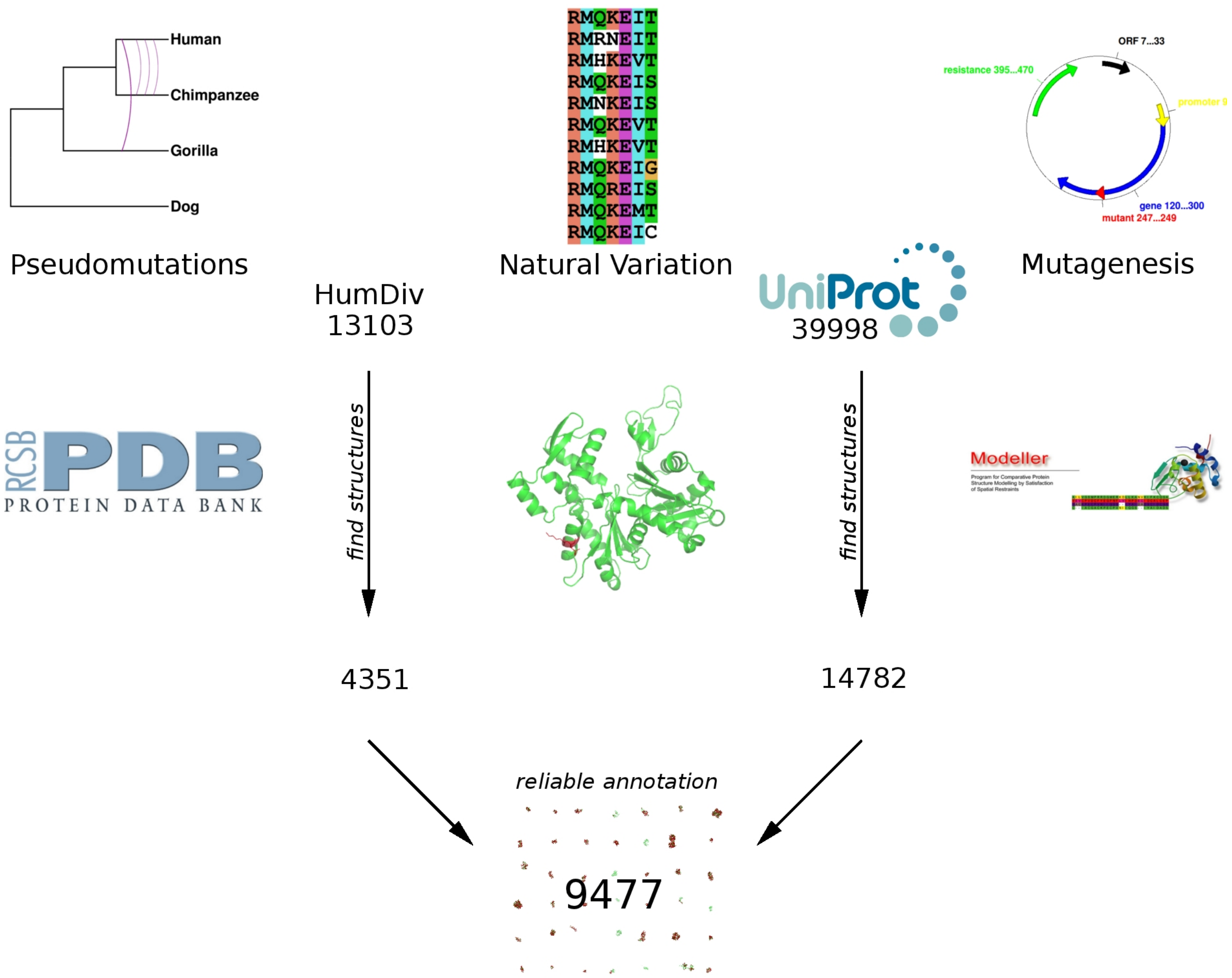

Supplementary Figure S1: **VTS Curation**: Variants with reliable annotations of deleterious or neutral effects on protein function are accumulated from UniProt and HumDiv. All variants must be mapped to structural models, limiting the number of useable annotations. Variants from all available species were extracted from UniProt reviewed entries and curated to include only reliable annotations of deleterious and neutral effects. Comparative models from ModBase and SwissModel are used when structural models are not available in the Protein Data Bank. The combined dataset reflects the diversity of variant data users are likely to use.

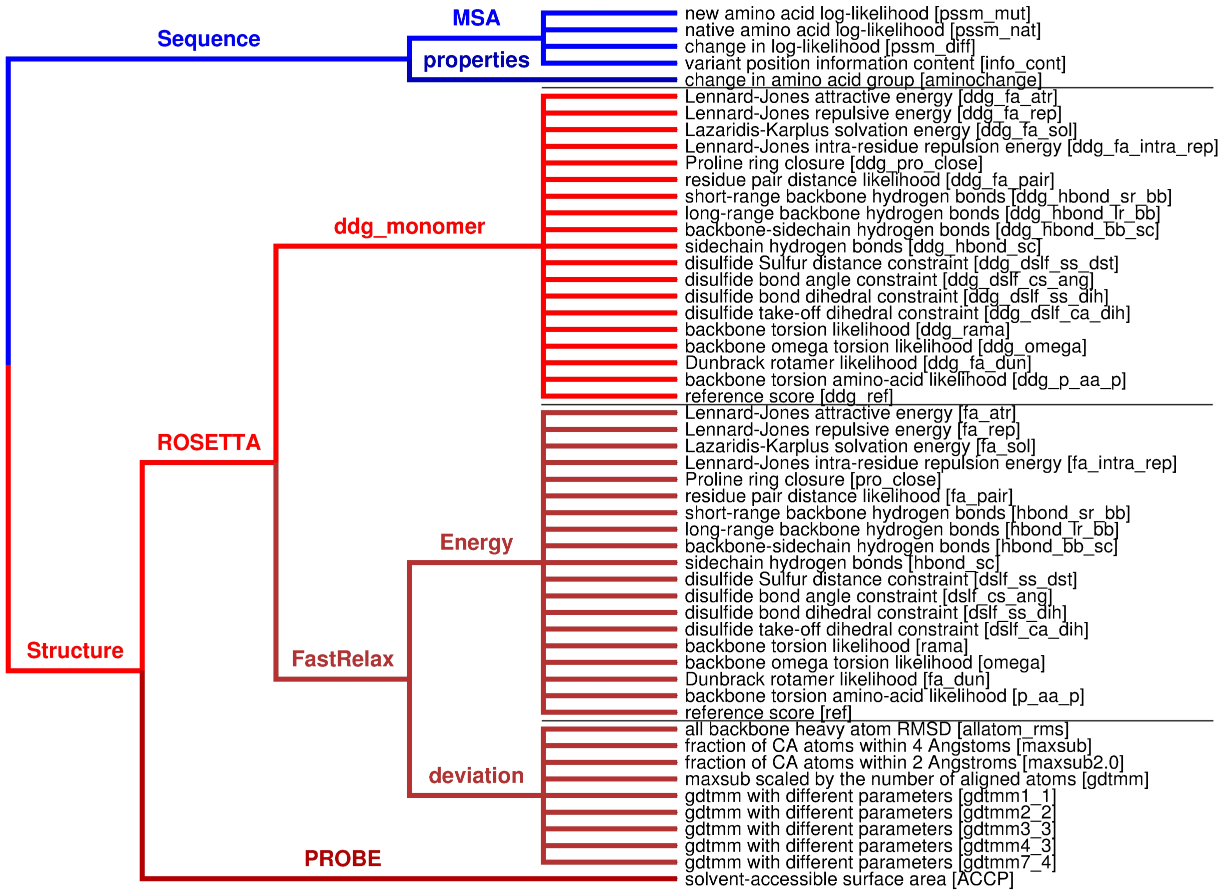

Supplementary Figure S2: **VIPUR Features**. A tree represents the conceptual hierarchy of all VIPUR features. Sequence-based features (blue) are extracted directly from a PSSM output by PSIBLAST, measuring the conservation and favorability of the amino acid substitutions. Structure-based features (red) are extracted from Rosetta simulations comparing the native and variant protein structures. Variant structures are refined using the `ddg_monomer` protocol and the more rigorous FastRelax protocol. Every branch listed for FastRelax will become three individual features for each variant, the quartiles (Q1, Q2, Q3) of the variant score distributions evaluated on the native score distribution. Two additional features approximate the change in accessible surface area (`ACCP`) and changes in amino acid properties (`aminochange`).

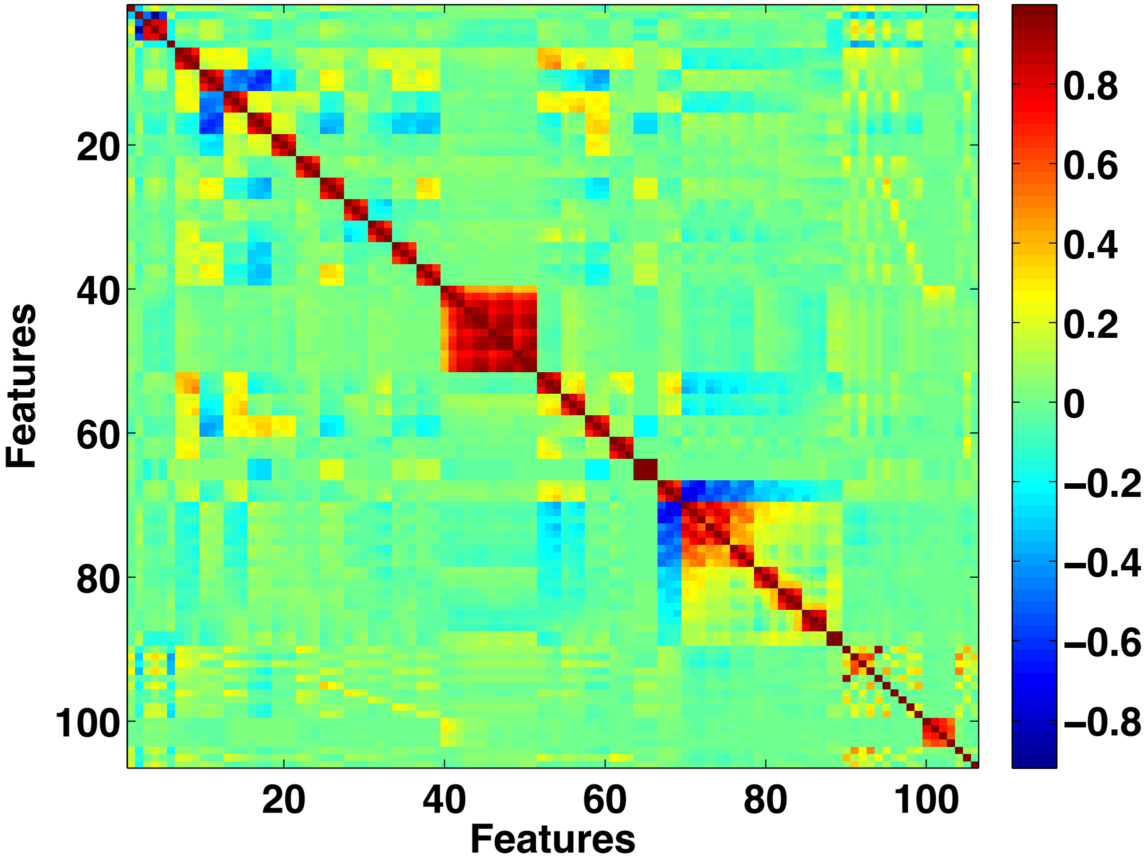

Supplementary Figure S3: **Pairwise Correlation Between Features**. Many of our features correlate highly with each other, suggesting a small subset of these features could represent the whole set with little loss. Several of these correlations are unsurprising. The three PSSM terms (features three, four, and five) contain similar information about amino acid conservation. Features derived from Rosetta FastRelax quartile analysis correlate highly with each other since three features (corresponding to the first, second, and third quartiles) are generated for each score term comparison between native and variant structures. There is also a strong correlation between the Rosetta disulfide scoring terms (blocks near the center and lower right) and gdtmm terms. Since many of these features are redundant, trained models with feature selection do not always converge on the same feature set.

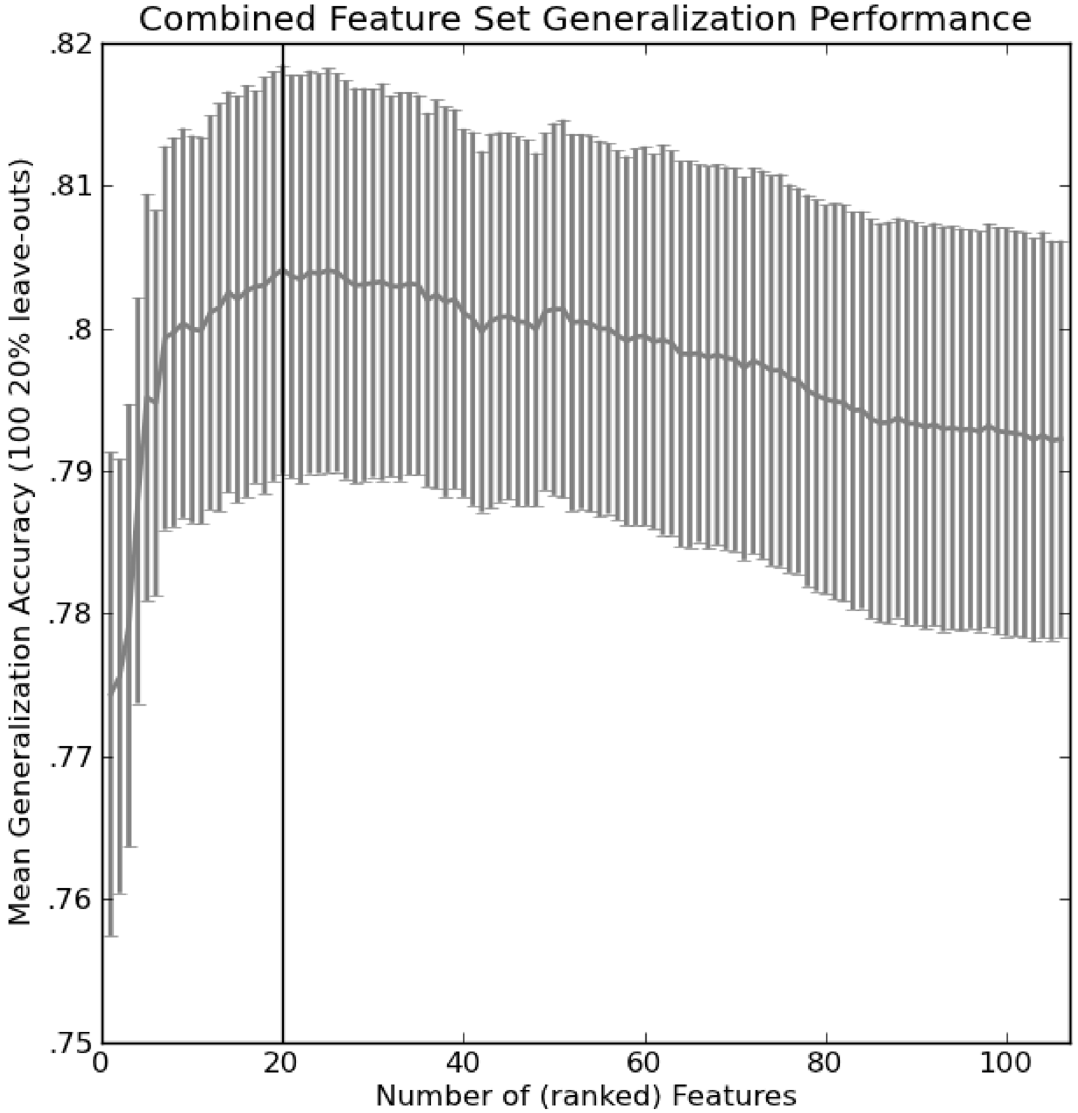

Supplementary Figure S4: **Feature Selection and Generalization**. Logistic regression models were trained with the entire feature set on 100 random splits using 80% of the data (each with optimized λ). Features are ranked by their occurrence in these models (left, frequency of selection) and generalization performance is tested by re-training models on these ranked feature sets (100 random splits using 80% of the data for training and 20% to test generalization). Generalization performance decreases when unnecessary features are included in the model (right). The first 20 features improve performance and are used in the final model for VIPUR. Generalization decreases when additional features are added since they provide redundant information, making the model sensitive to fluctuations in the training set (overfit).

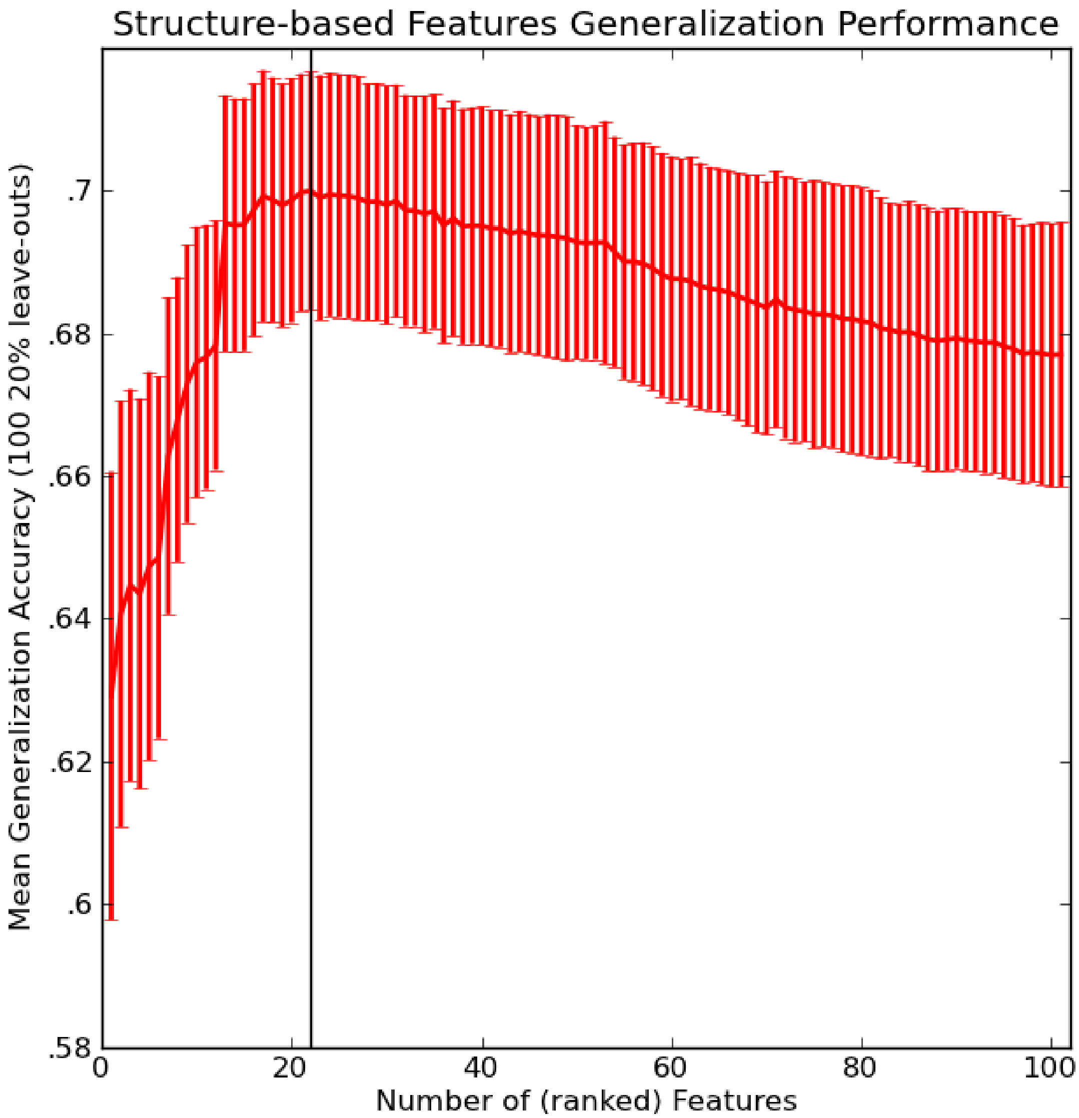

Supplementary Figure S5: **Structure-only Feature Selection and Generalization**. Logistic regression models were trained with just the structure-based features on 100 random splits using 80% of the data (each with optimized λ). Structure-based features are ranked by their occurrence in these models (left, frequency of selection) and generalization performance is tested by re-training models on these ranked feature sets (100 random splits using 80% of the data for training and 20% to test generalization). Generalization performance decreases when unnecessary features are included in the model (right). For the structure-based features, the first 22 features improve performance. Generalization decreases when additional features are added since they provide redundant information, making the model sensitive to fluctuations in the training set (overfit).

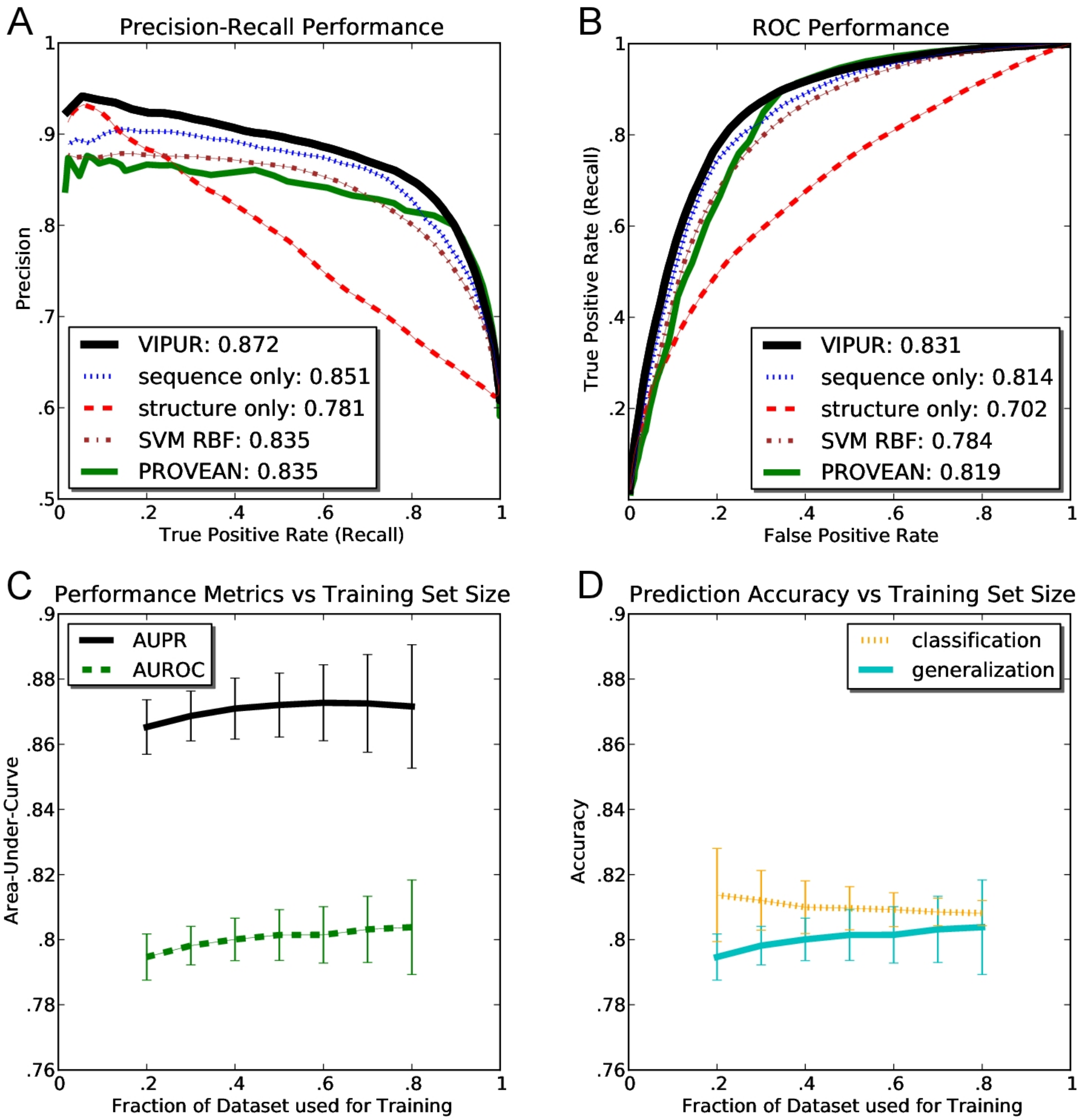

Supplementary Figure S6: **ROC and PR Curves**. Averaged from models trained on 100 random dataset splits (80% training, 20% testing). Performance of PROVEAN (green) and a Radial Basis Function Support Vector Machine (brown) classifier are shown for comparison. A) VIPUR achieves a higher AUPR than other methods due to the enhanced precision of structure-based features. Sequence based methods (PROVEAN and our sequence-only classifier) generalize well, but cannot indicate confident predictions. VIPUR's confidence score scales with precision, clearly indicating which predictions are more likely to be correct. B) ROC performance similarly shows that VIPUR is more specific than other methods at high confidence scores. PROVEAN and our sequence-only classifier, clearly indicate this sensitivity-specificity tradeoff. C) These model properties are robustly produced during training and do not change much when trained on more than 50% of our training set. D) Classification and generalization (leave-out) performance converge for our feature set, indicating our model is not overfit and performance estimates are reliable.

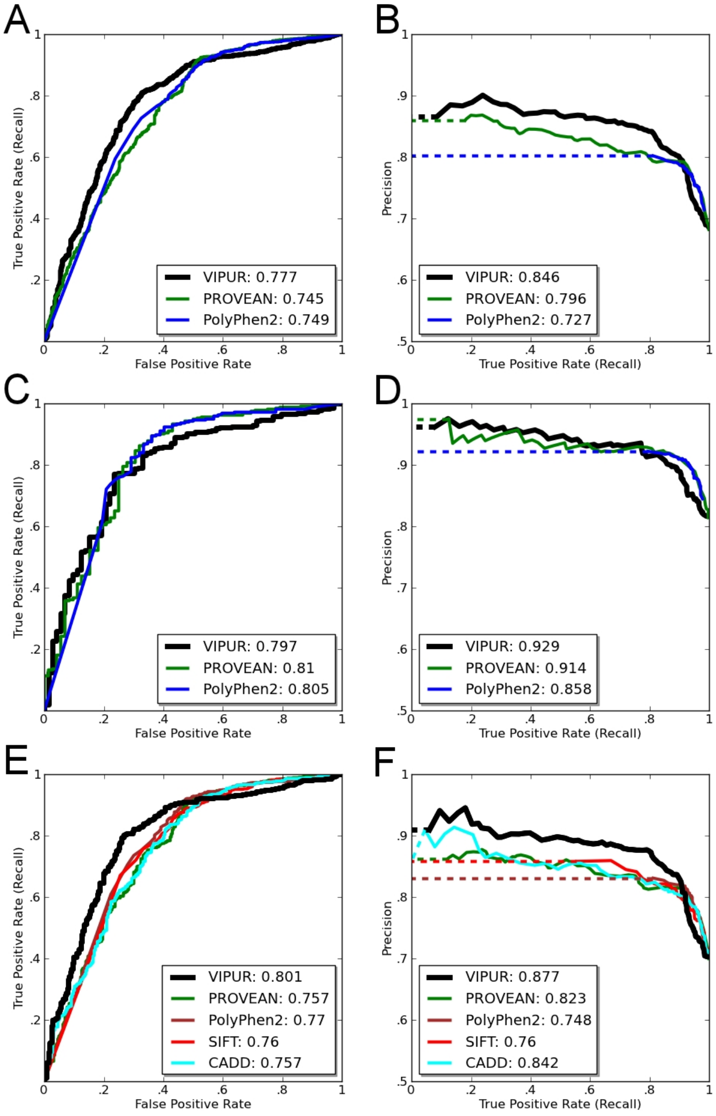

Supplementary Figure S7: **ROC and PR Curves on Human Variants**. AUROC and AUPR curves are calculated for a set of human variants in **VTS** (1,542 variants). These variants are not contained within HumDiv and A VIPUR classifier is retrained to exclude these variants, allowing the VIPUR feature space to be compared with PolyPhen2. A) VIPUR has a increased AUROC compared to PROVEAN and PolyPhen2, with notably higher AUPR (B), indicating VIPUR's top predictions are more enriched for true deleterious variants compared to other methods. C, D) PROVEAN and PolyPhen2 have notably improved performance on common human variants (383 variants), suggesting these methods may be overfit to this type of variation. VIPUR still attains a higher AUROC on these natural variants with a dramatically higher AUPR. E,F) SIFT and CADD predictions are available only on a subset of these human variants (950 variants) and have curves similar to PolyPhen2 and PROVEAN on the 1,542 human variant set. VIPUR performs better than all other methods tested, although CADD's ranking ability is the only method to deviate noticeably from the other methods.

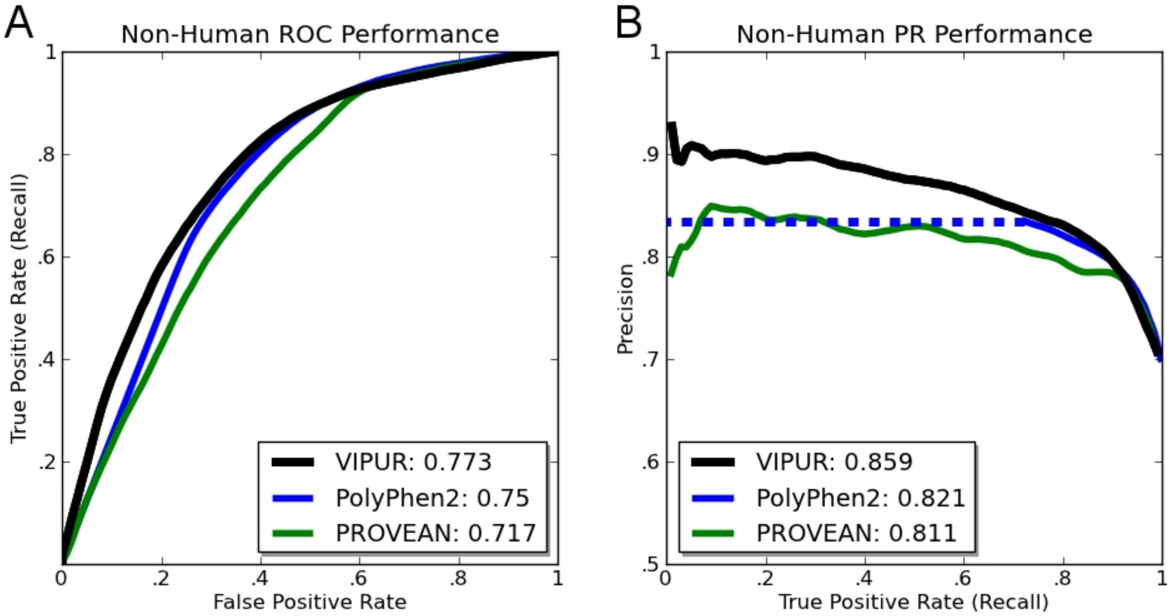

Supplementary Figure S8: **ROC and PR Curves on Non-Human Variants**. AUROC and AUPR curves are calculated for a set of variants from non-human species in **VTS** (4,992 variants). ROC (A) and PR (B) Curves are calculated on 100 random samples of 1,000 variants in this set. VIPUR achieves higher AUROC and AUPR than the other methods.PolyPhen2 and PROVEAN perform very well considering they are not trained on any variants found in species other than humans. All three methods perform worse on these variants than human variants, however VIPUR's AUPR is consistently $\geq .85$ in this and other comparisons.

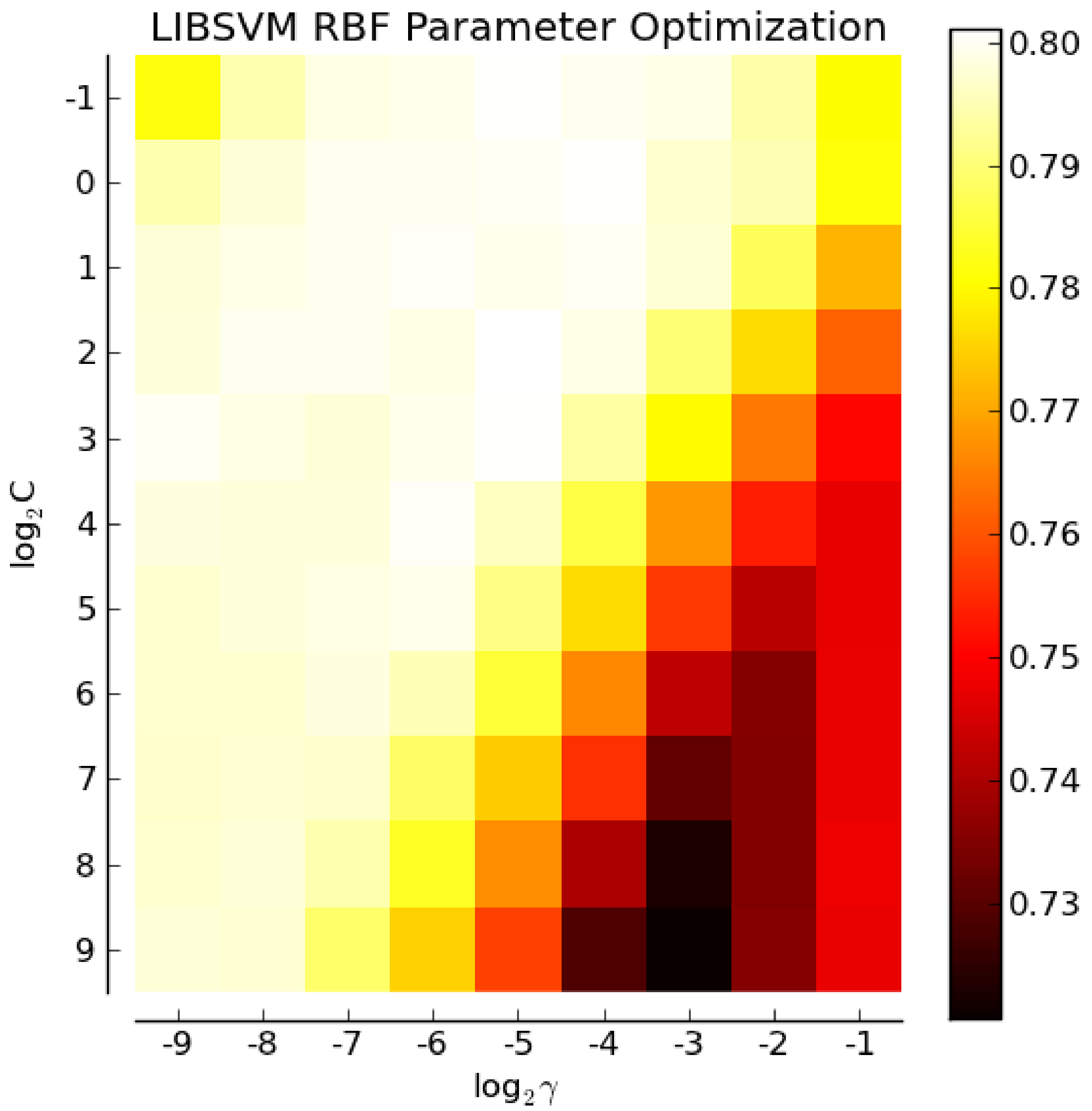

Supplementary Figure S9: **Parameter Optimization for a Radial-Basis Function Support Vector Machine using LibSVM**. Optimum values for the meta parameters C (SVM cost) and γ (RBF variance) were chosen by training over $C \in \{2^{-1}, 2^0, 2^1, ..., 2^9\}$ and $\gamma \in \{2^{-8}, 2^{-7}, ..., 2^{-1}\}$. The values (C= 2^2, $\gamma = 2^{-4}$) maximize accuracy using 5-fold cross-validation (highest average accuracy on the leave-out sets).

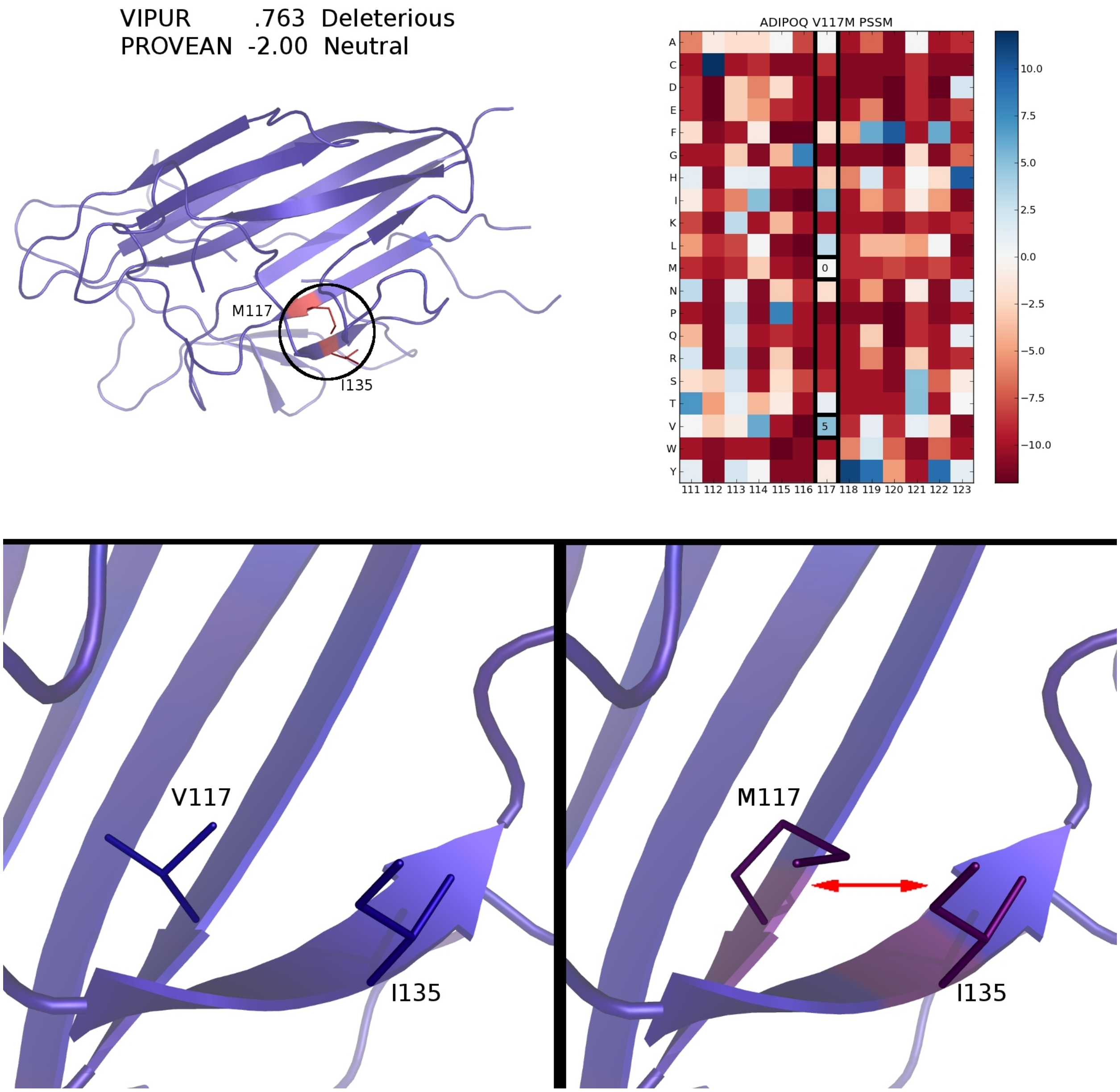

Supplementary Figure S10: **V117M Disrupts Strand Pairing for ADIPOQ**. V117M is predicted deleterious (.763) due to statistically unfavorable backbone conformation and predicted neutral (-2.00) by PROVEAN. Every residue in ADIPOQ is colored by the difference in Rosetta energy between the native and variant protein structures, highlighting the destabilization introduced by V117M (top left). The PSSM generated by PSIBLAST does not indicate strong conservation at position 117 (top right, PSSM columns shown for surrounding residues). The native V117 structure forms stable hydrophobic contacts and strand pairing (bottom left, residues colored by Rosetta energy of a representative model). The M117 variant model cannot accommodate the larger amino acid without disrupting strand pairing.

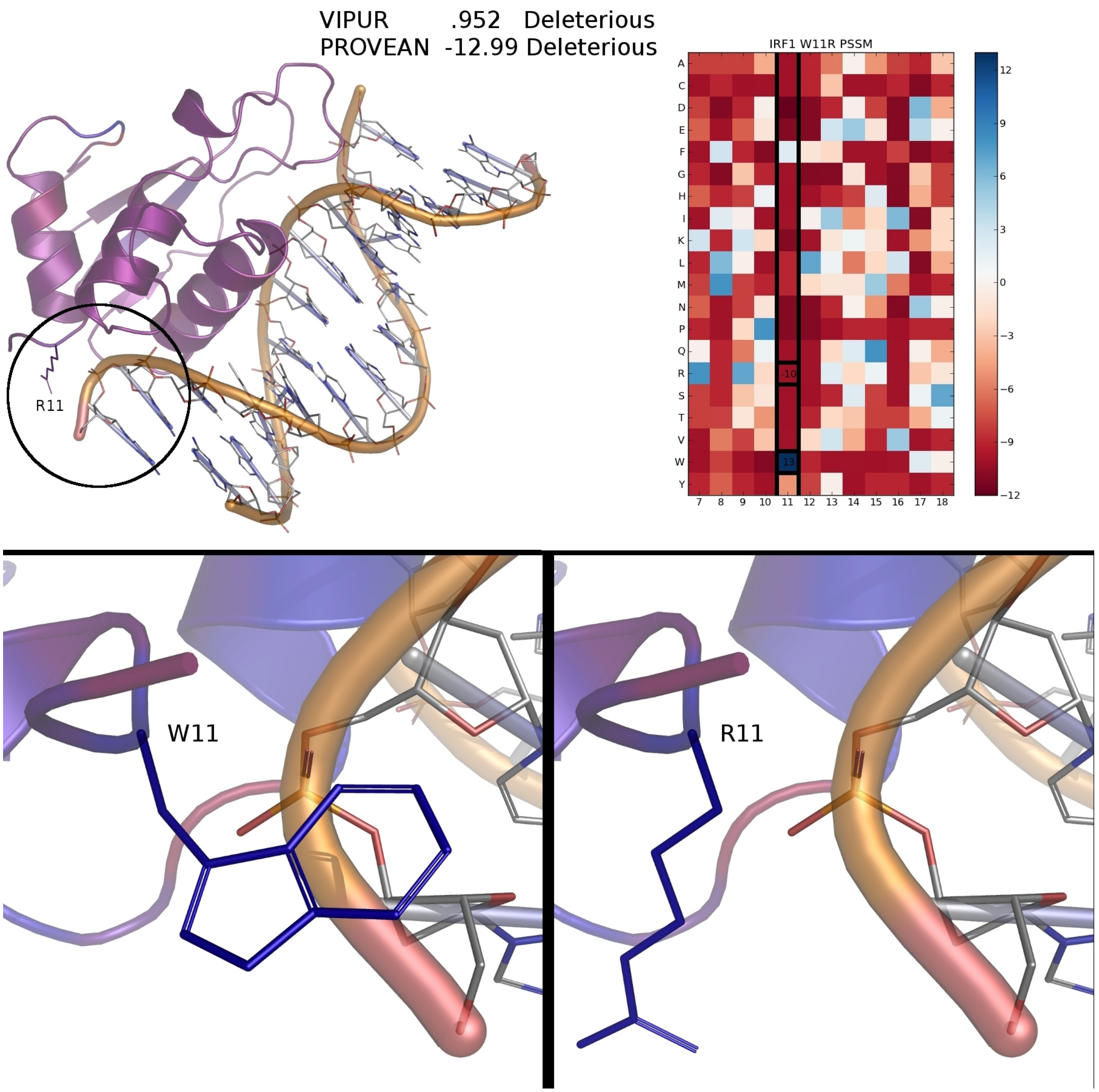

Supplementary Figure S11: **W11R Eliminates a DNA Contact**. VIPUR predicts W11R is deleterious (.952), matching the UniProt annotation that this variant “abolishes DNA binding”. Our structural model lacks DNA and the correct prediction is due to the sequence-based features. Every residue in IRF1 is colored by the difference in Rosetta energy between the native and variant protein structures, demonstrating the similarity of energies between native and variant structures (top left). The PSSM generated by PSIBLAST indicates that W11 is highly conserved (top right, PSSM columns shown for surrounding residues). W11 packs against the DNA backbone in the template PDB 1IF1 (bottom left, residues colored by Rosetta energy of a representative model) and this contact is lost in R11 (bottom right). Without DNA in the structural model, W11R is not detected as energetically destabilizing, however, the sequence-based features accurately inform a deleterious prediction.

2,295 variants from ClinVar (1,797 path, 498 ben, 3.61 label bias)

method	path-del	path-neu	ben-del	ben-neu	path enrich	ben enrich
VIPUR	1,340	457	120	378	11.17	1.21
PolyPhen2	1,590	207	177	321	8.98	0.64
PROVEAN	1,564	233	132	366	11.85	0.64
SIFT	1,509	288	148	350	10.20	0.82
CADD	1,587	210	126	372	12.60	0.56
BLOSUM62	1,430	367	312	186	4.58	1.97

Supplementary Table S6: ClinVar Predictions
path-del: pathogenic variants in deleterious predictions (True Positives) at .5 cutoff, prb-neu: pathogenic variants in neutral predictions (False Negatives) at .5 cutoff, ben-del: benign variants in deleterious predictions (False Positives) at .5 cutoff, ben-neu: benign variants in neutral predictions (True Negatives) at .5 cutoff, path enrich: the ratio of path-del/ben-del, ben enrich: the ratio of path-neu/ben-neu

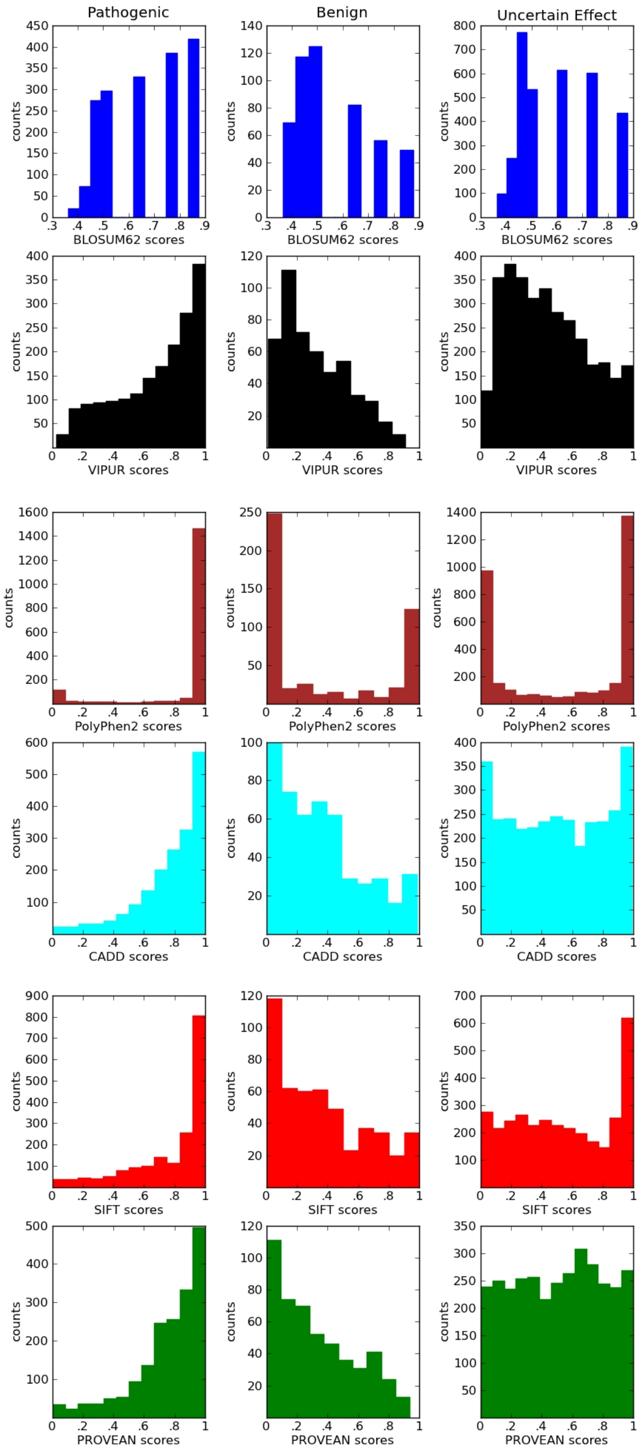

Supplementary Figure S12: **Pathogenic, Benign, and Uncertain Effect Variant Score Distributions**. Score histograms for several methods on ClinVar pathogenic, benign, and uncertain variants. All methods match expectations for pathogenic and benign variants, with pathogenic variants having a skewed distribution of deleterious scores and benign variants having a broad distribution of neutral scores. PolyPhen2 predictions are notably pushed to high and low values. Predictions on variants with uncertain labels are very diverse and suggest very different error rates between available methods.

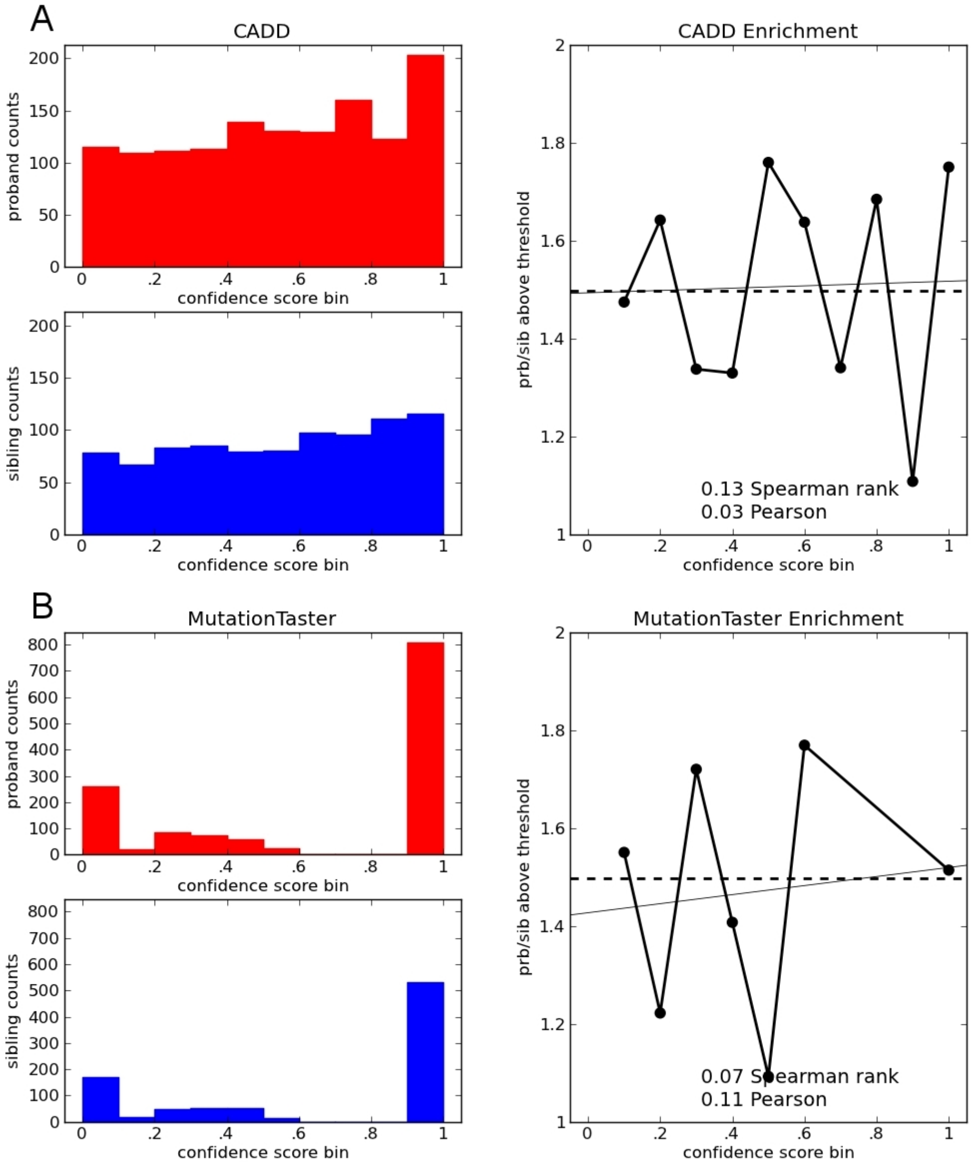

Supplementary Figure S13: **Enrichment for ASD-Associated De novo Variation by Additional Methods**: Predictions for various methods on the Simons Simplex Collection, containing 1,335 *de novo* mutations found in children with autism-spectrum disorders (probands) and 891 *de novo* mutations found in unaffected siblings. For each prediction method, the distribution of confidence scores is shown for proband (red) and sibling (blue) mutations. The ratio of these counts for each score bin are shown along with the background expectation (dashed line, 1.50, 1,335/891). We expect high deleterious scores to be enriched for proband mutations and low scores enriched for mutations found in siblings. A) CADD predictions do not noticeably enrich for proband or sibling mutations. The highest CADD scores are enriched for proband mutations, but not noticeably more that other score bins, leading to a negligible correlation. B) MutationTaster effectively splits mutations into high and low confidence bins with very few mutations scoring in between. This top bin is not strongly enriched for proband mutations leading to a negligible correlation.

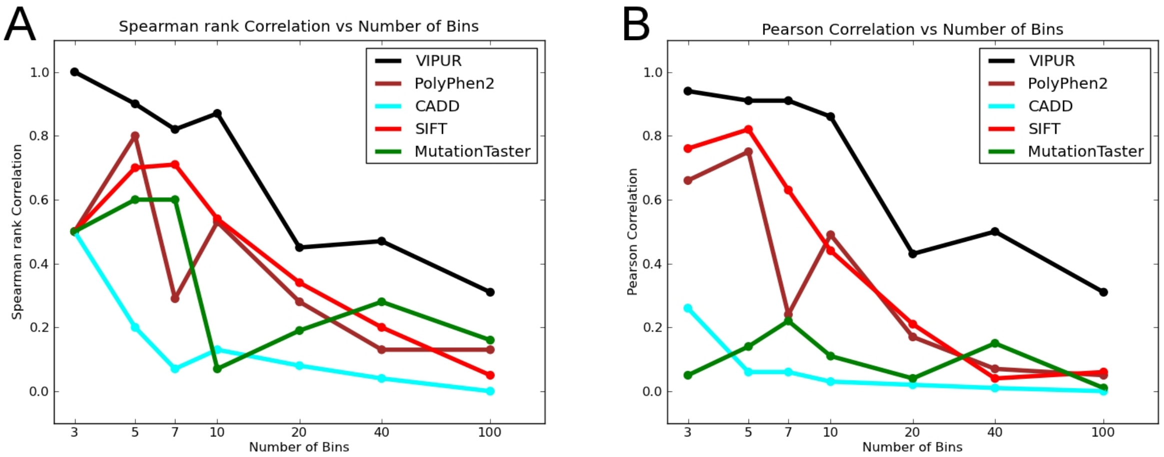

Supplementary Figure S14: **Simons Simplex Collection Proband Enrichment is Robust to the Bin Size**. Altering the number of bins used for the correlation calculation between deleterious scores and proband enrichment changes the correlation value obtained but not the trend. These methods predict categorical labels from a continuous deleterious score where low values indicate neutral mutations and high values indicate highly disruptive mutations. VIPUR predictions produce higher correlation values than other pathogenicity prediction methods independent of the number of bins used for the calculation.

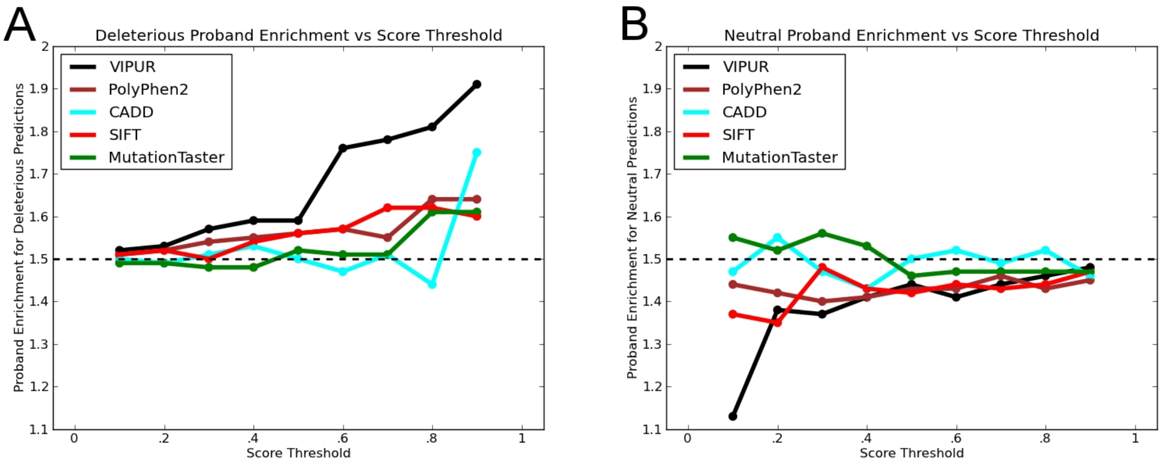

Supplementary Figure S15: **Simons Simplex Collection Analysis Proband Enrichment is Robust to the Score Threshold**. Altering the threshold used to determine deleterious vs neutral predictions changes the enrichment ratio obtained but does not notably alter the trend. This evaluation is the accumulated version (accumulated to 1 for deleterious predictions and to 0 for neutral predictions) of the bin correlation (Figure 6). VIPUR predictions are consistently above the background ratio for deleterious predictions and below the background ratio for neutral predictions, with sharp increases for high confidence predictions. PolyPhen2 and SIFT both maintain the appropriate trends but are have consistently worse enrichment ratios compared to VIPUR.
